# Evaluating Artificial Intelligence for Sepsis Prediction in Emergency Departments: A Systematic Review and Meta Analysis

**DOI:** 10.1007/s10916-026-02376-3

**Published:** 2026-04-13

**Authors:** Yinan Zhang, Tim Kirchler, Audrey P. Wang

**Affiliations:** DHI Lab, Biomedical Informatics and Digital Health, Sydney School of Public Health, The University of Sydney, Westmead, NSW 2145 Australia

**Keywords:** Sepsis, Artificial intelligence, Prediction models, Emergency medical services, Clinical decision support system

## Abstract

This study aims to synthesize current evidence on artificial intelligence-based sepsis prediction models for emergency department patients and propose practical benchmarks that emphasize standardized data preparation and reproducible model characterization. Literature searches were conducted across Scopus, Web of Science, PubMed, MEDLINE, and Embase. Eligible studies were selected through a two-tiered screening process, followed by data extraction and assessment according to predefined criteria. Random-effects meta-analysis was used to quantify model performance, and heterogeneity was explored by subgroup, regression, and sensitivity analyses. A total of 36 studies comprising 98 predictive models were included, with a pooled area under the receiver operating characteristic curve of 0.87 (95% CI: 0.86–0.88). Differences in performance were associated with study-level methodologies, including target definition, data provenance, cohort scale, data preprocessing, feature representation, and model development. The integrated meta-regression further identified independent methodologies influencing model performance. Artificial intelligence-based models showed higher pooled predictive performance than widely used traditional scoring systems for sepsis in emergency departments. However, translation into practice remains limited by inconsistent evaluation and reporting, and by inadequate external validation. Standardized methodological benchmarks have the potential to improve reproducibility, comparability, and clinical applicability.

**Supplementary Information:**

The online version contains supplementary material available at 10.1007/s10916-026-02376-3.

## Introduction

Sepsis is a life-threatening condition indiscriminately affecting individuals of all ages, defined by the Sepsis-3 criteria as organ dysfunction caused by a dysregulated host response to infection [1]. Data released in 2020 showed that approximately 48.9 million sepsis cases occurred worldwide, leading to 11 million deaths, estimated to represent around 20% of global mortality [2]. Clinical management of sepsis is time-sensitive, with mortality risk escalating by 4–7% for each delayed hour in antibiotic administration [3, 4]. In emergency departments (EDs), early sepsis recognition is limited due to rapid turnover, resource constraints, high cognitive loads [5, 6], and different patient presentations [7–9], risking delayed interventions and adverse outcomes.

Recent studies suggest that artificial intelligence (AI) increasingly outperforms rule-based screening tools such as Systemic Inflammatory Response Syndrome (SIRS), quick Sequential Organ Failure Assessment (qSOFA), and Modified Early Warning Score (MEWS) [10]. Traditional approaches have several limitations in spite of widespread clinical adoption, including insufficient sensitivity and specificity due to oversimplified thresholds [11], delayed responsiveness stemming from intermittently manually entered data [12], and limited adaptability across diverse patient groups [13]. By comparison, AI technologies have the potential to overcome these difficulties through real-time analysis of clinical datasets to detect physiological trajectories indicative of early-stage sepsis [12]. For example, a multicentre study successfully predicted 80% of septic cases an average of 3.7 h before onset using international databases [14]; and another triage-level analysis with over one million participants leveraged natural language processing to achieve an area under the receiver operating characteristic curve (AUROC) of 0.94 [15]. Integration of such models facilitates earlier alerts and intensive patient monitoring, helping EDs to mitigate diagnostic errors and mortality risks [16, 17].

However, methodological variability and inconsistent results across studies limit comparability and generalizability. It remains unclear under which conditions AI-based models offer robust advantages over conventional warning systems across different healthcare contexts and patient demographics. Some evidence indicates superior AI performance primarily under optimal conditions, including extensive, high-quality datasets and higher prevalence [18, 19]. Conversely, others report limitations arising from algorithm biases, incomplete clinical annotations, and inadequate external validations [20, 21]. Thus, systematically evaluating AI effectiveness in EDs and establishing methodological guidelines are necessary steps toward bridging these gaps.

Existing reviews on sepsis prediction focus primarily on isolated technologies and rarely conduct comprehensive analyses of model development. Previous studies discussed diagnostic ‘gold standard’ definitions [14, 22–25], data processing strategies [23, 26], predictive features [22, 24–31], algorithm selection [22, 23, 26, 29, 32, 33], and evaluation indicators [29, 31, 34]. Nevertheless, the absence of standardized evaluation criteria and cohesive modeling benchmarks complicates comparative analysis, undermines clinical interpretability, and limits actual adoption. Similarly, overlooking clarity in data preparation pipelines and consistency in model development may lead to performance biases, yielding high accuracy in controlled experiments but reduced performance in unfamiliar scenarios.

**Fig. 1:**
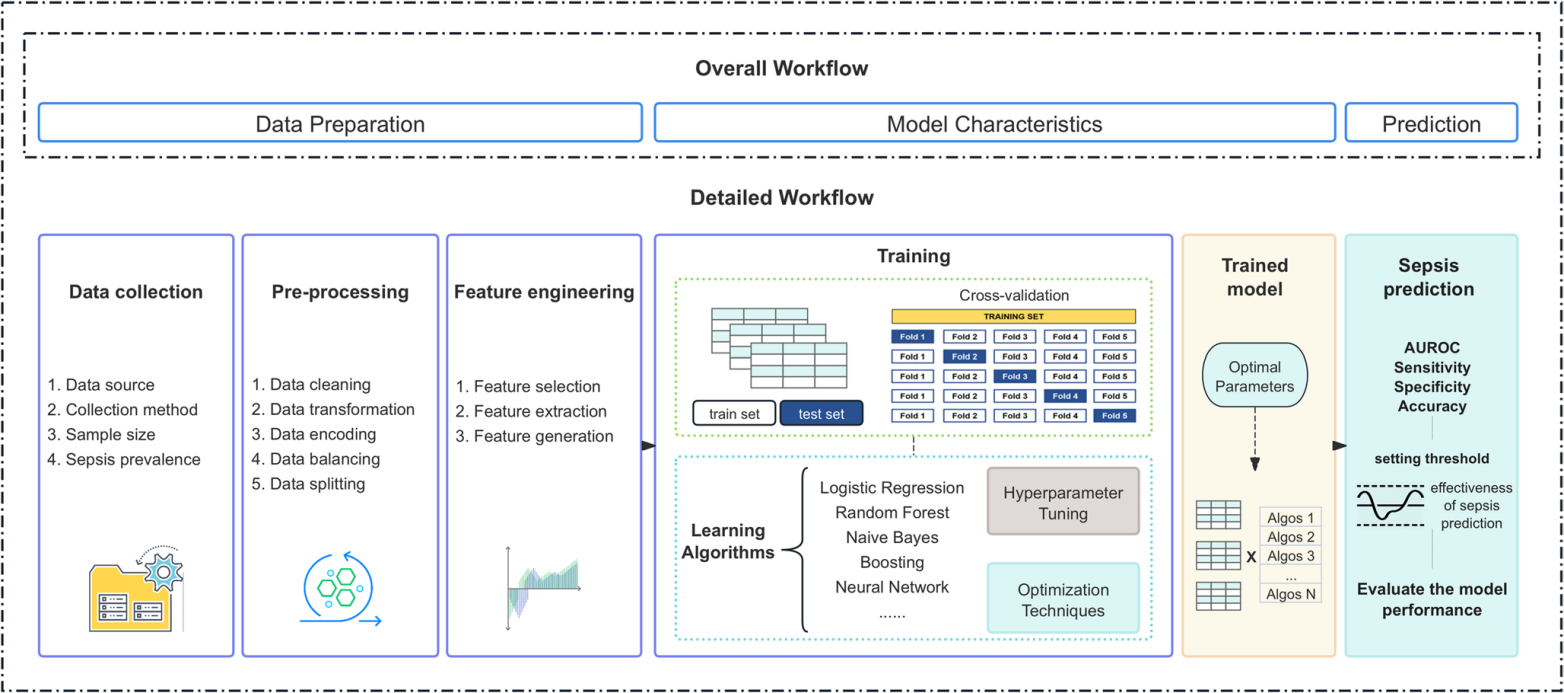
Overall workflow for sepsis prediction models

Therefore, this review aims to evaluate recent AI-based sepsis prediction models in EDs with a focus on data preparation and model characteristics (Fig. 1). Theoretically, it provides a methodological benchmark to promote standardized and reproducible modeling approaches. Practically, it offers evidence-based recommendations to develop AI-enhanced clinical decision support systems, ensuring feasibility and effective integration into emergency medical workflows. The review is guided by three specific research questions:

1. How effectively do AI-based methods predict sepsis in EDs compared with traditional assessment tools?
2. How can credible, generalizable performance be maintained or enhanced through data preparation and model characterization?
3. How can standardized evaluation and reporting support reproducibility, cross-study comparability, and readiness for clinical implementation?

## Methods

This systematic review and meta-analysis adheres to the Preferred Reporting Items for Systematic Reviews and Meta-Analyses (PRISMA) guidelines [35]. The protocol has been pre-registered with the Open Science Framework (OSF) (osf.io/jkbr5).

### Search Strategy

On November 8, 2024, an exhaustive search was conducted across five databases: Scopus, Web of Science, PubMed, MEDLINE, and Embase. The search strategy encompassed four primary concepts: ‘Sepsis’, ‘Artificial Intelligence’, ‘Emergency Department’, and ‘Prediction’ (see Table S1). Search terms included subject headings (MeSH in PubMed and MEDLINE, Emtree in Embase), along with synonyms and alternatives. These terms were orchestrated using Boolean operators, and their order of operation was grouped with parentheses (e.g., ‘Sepsis AND (Diagnosis OR Prognosis)’). Field-specific tags guided the searches within designated fields, while exact phrases were constructed using double quotes (curly brackets { } instead in Scopus). An asterisk ‘*’ functioned as a wildcard for truncation searches, accommodating variations of the root word (e.g., ‘Predict*’ could cover ‘Predicts’, ‘Predicting’ or ‘Prediction’). Additionally, searches were constrained to journals published from 2019 onwards to obtain recent developments in AI and excluded review-type articles. Detailed query strings are provided in Supplementary File S1.

### Study Screening and Eligibility Criteria

Retrieved records were imported into EndNote 21.2 for duplication and managed using Covidence. Two researchers (YZ and TK) independently performed a two-tiered screening to minimize biases. Inclusion criteria comprised studies that: (1) were peer-reviewed journal articles to mitigate the unreviewed bias; (2) were published in English for accessibility; (3) were published from 2019 onwards to ensure the latest development of AI technology; (4) explicitly mentioned sepsis prediction in titles or abstracts; (5) applied AI algorithms (e.g., Machine Learning or Deep Learning), and highlighted in titles or abstracts; (6) were conducted in emergency department facilities instead of non-ED or outpatient settings [36]; (7) reported predictive model effectiveness measures. We excluded case reports and review articles, as well as studies focused on non-human subjects or experimental research unrelated to human healthcare.

Following a pilot screening of 100 publications, titles and abstracts were reviewed against predefined eligibility criteria (Table S2 and S3) to exclude obviously irrelevant studies. Full-text evaluations were subsequently conducted on candidate articles to determine eligibility for inclusion. Any discrepancies between two reviewers were resolved by consensus, with arbitration by the third reviewer (AW) when necessary. Screening processes were well-documented and reported using a PRISMA flow diagram, clearly outlining rationales for exclusion during the full-text phase.

### Data Extraction

A standardized data extraction form was designed and applied to all included studies, structured around three domains: study details (Table S4), data preparation (Table S5), and model characteristics (Table S6). The extraction process was fully documented to support transparency and reproducibility.

Extracted information included: (1) study details: authors, year of publication, country, clinical setting, demographics, sepsis definition, clinical comparator, research objective, study period; (2) data preparation: data source, collection method, sample size, training/validation/testing datasets, positive patients, sepsis prevalence, data preprocessing methods, feature engineering, feature numbers (initial and final), feature types, feature importance; (3) model characteristics: algorithms, optimization methods, AUROCs, other output metrics, accuracy, sensitivity, specificity, prediction window, effectiveness summary.

### Quality Assessment

The quality assessment was conducted based on a predefined TRIPOD + AI framework to evaluate the reporting completeness and transparency of included studies [37], which was adapted specifically for this review. Each study was rated ‘YES’, ‘NO’, or ‘Not Applicable (N/A)’ across 52 items organized into five sections: introduction, methods, open science, results, and discussion (details are shown in Table S7).

### Statistical Analysis

Descriptive statistics summarized key characteristics of included studies in terms of research objectives, publication years, countries, patient demographics, modeling features and algorithm types. Between-study heterogeneity was evaluated using the $\:{I}^{2}$ statistic. Pooled performance metrics (AUROC, accuracy, sensitivity, and specificity) and their 95% confidence intervals were estimated using random-effects meta-analysis.

Subgroup analysis was performed stratified by prediction outcomes, sepsis definitions, data sources, collection methods, sample sizes, sepsis prevalence, feature numbers, dataset divisions, and algorithm types. Univariate and multivariate meta-regressions were fitted to examine variables influencing sepsis model performance and sources of heterogeneity. An integrated model was further used to estimate associations between candidate variables and model performance, and to quantify heterogeneity explained. Its inference was based on study-clustered robust standard errors to account for dependence among multiple effect sizes reported in the same study, thus yielding more conservative uncertainty than conventional model-based tests. All regression models accounted for within-study clustering of multiple reported models by including study-level random effects. Besides, the leave-one-out sensitivity analysis systematically excluded individual studies to test result robustness, and Egger’s funnel plot was used for assessing publication biases.

Considering that only AUROC was universally reported across studies, it was selected as the primary measure. Since AUROC values were bounded between 0 and 1, a logit transformation was applied prior to regression analyses to stabilize variance and satisfy statistical assumptions. For clarity, factors used for model development were defined as ‘predictors’ and those used for study-level regression analyses as ‘variables’. All analytical procedures were conducted using R software version 4.4.2.

## Results

### Study Selection

**Fig. 2:**
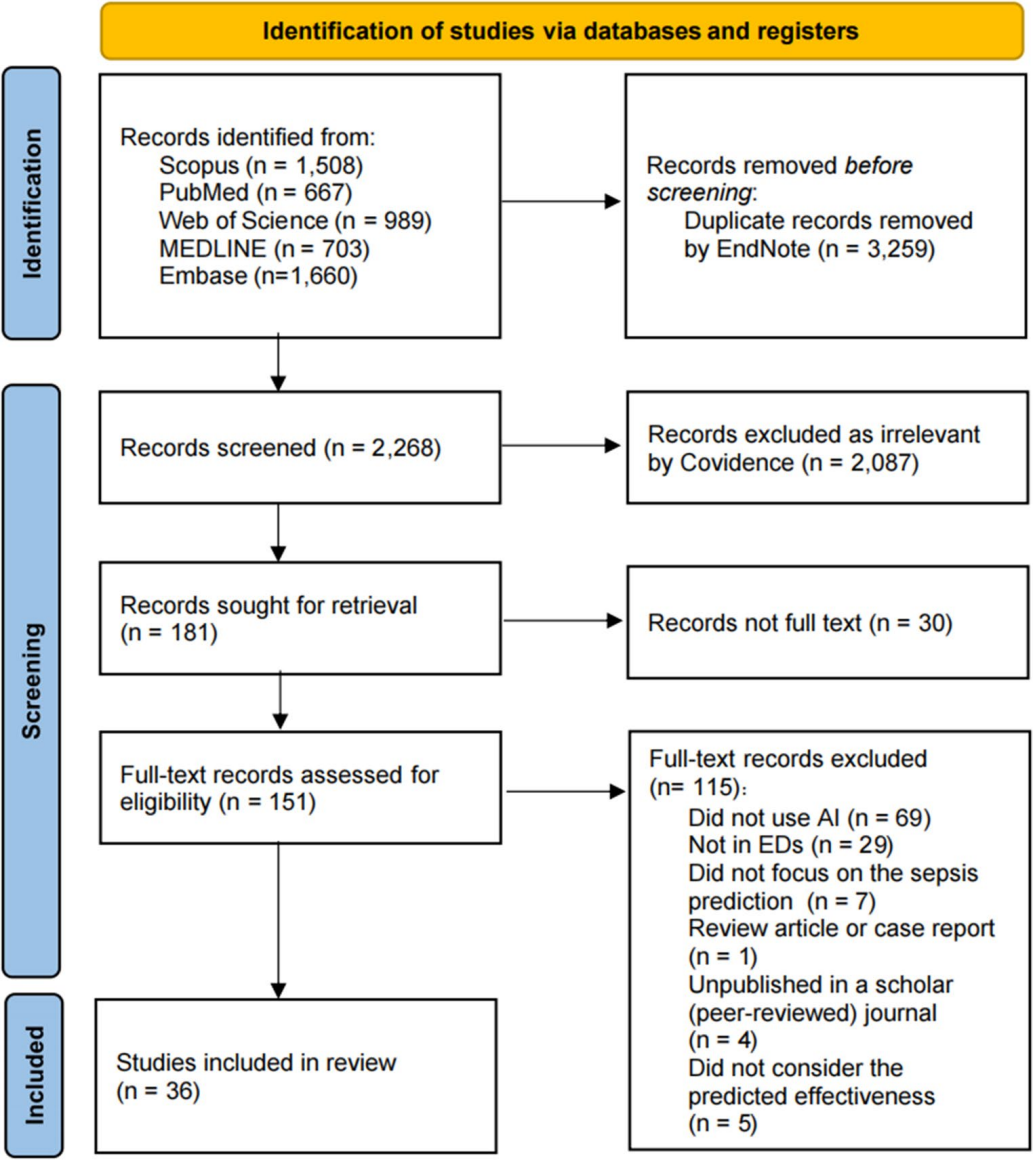
PRISMA flow diagram of the study screening

The data screening process is summarized in a PRISMA flowchart (Fig. 2). Initially, 5,527 records were retrieved from five databases and imported into EndNote, with 3,259 duplicates subsequently removed. Covidence facilitated the screening of remaining records, excluding 2,087 irrelevant titles and abstracts, leaving 181 articles for full-text review. 30 reports were not retrieved in full text because they were conference abstracts/records only, even after attempts to locate related full publications by the same authors. Ultimately, 36 studies satisfied the inclusion criteria. The main reasons for exclusion were the absence of AI tools or non-ED settings.

### Study Characteristics

The included studies (*n* = 36) were observational cohort studies primarily aimed at prediction of early-onset sepsis (*n* = 19, 53%) [15, 38–55], sepsis-related mortality (*n* = 11, 31%) [43, 56–66], septic shock (*n* = 5, 14%) [67–71], and sepsis prognosis (*n* = 1, 3%) [72]. The majority originated from the U.S. healthcare system (*n* = 12, 33%) and focused on adult patient visits (*n* = 29, 81%). Frequently employed AI algorithms included boosting models (*n* = 19, 53%), logistic regression (LR) (*n* = 17, 47%), random forest (RF) (*n* = 16, 44%), and neural network (NN) (*n* = 15, 42%).

**Fig. 3:**
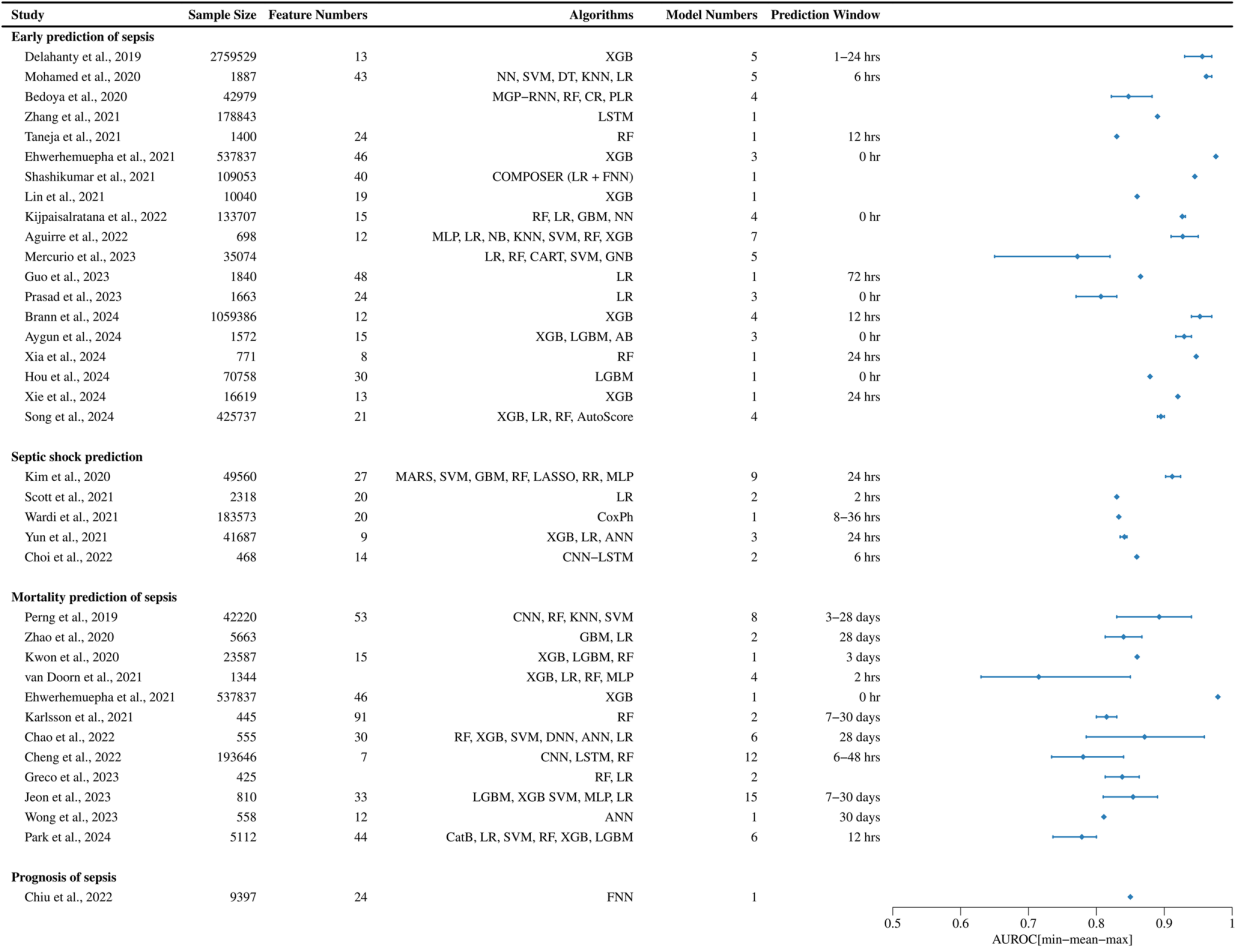
Overview of included studies. *AB=*Adaptive Boosting*, ANN=*Artificial Neural Network*, CR=*Cox Regression*, CART=*Classification and Regression Tree*, CatB=*Categorical Boosting*, CNN=*Convolutional Neural Network,* CoxPh=*Cox Proportional Hazards*, DT=*Decision Tree*, DNN=*Deep Neural Network*, FNN=*Feedforward Neural Network*, GBM=*Gradient Boosting Machine*, GNB=*Gaussian Naive Bayes*, KNN=*K-Nearest Neighbors*, LGBM=*Light Gradient Boosting Machine*, LSTM=*Long Short-Term Memory*, LASSO=*Least Absolute Shrinkage and Selection Operator*, MLP=*Multi-layer Perceptron*, MARS=*Multivariate Adaptive Regression Splines*, MGP=*Multi-output Gaussian Process*, NB=*Naïve Bayes*, PLR=*Penalized Logistic Regression*, RNN=*Recurrent Neural Network*, RR=*Ridge Regression*, SVM=*Support Vector Machine*, XGB=*Extreme Gradient Boosting

AUROC values were model-level results reported in included studies and were presented descriptively by prediction outcomes, shown with sample sizes, feature and model numbers, algorithm types, and prediction windows (Fig. 3, adapted from Fleuren et al. [25], extracted from Table S4–6). AUROCs ranged from 0.65 to 0.98 for early-onset prediction, 0.83 to 0.92 for septic shock and prognosis, and 0.63 to 0.98 for mortality prediction. Prediction windows were generally within 24 hours for sepsis onset and shock, whereas they spanned up to 30 days for mortality. Sample sizes varied widely (< 500 to > 2 million), with models incorporating up to 91 features.

**Fig. 4:**
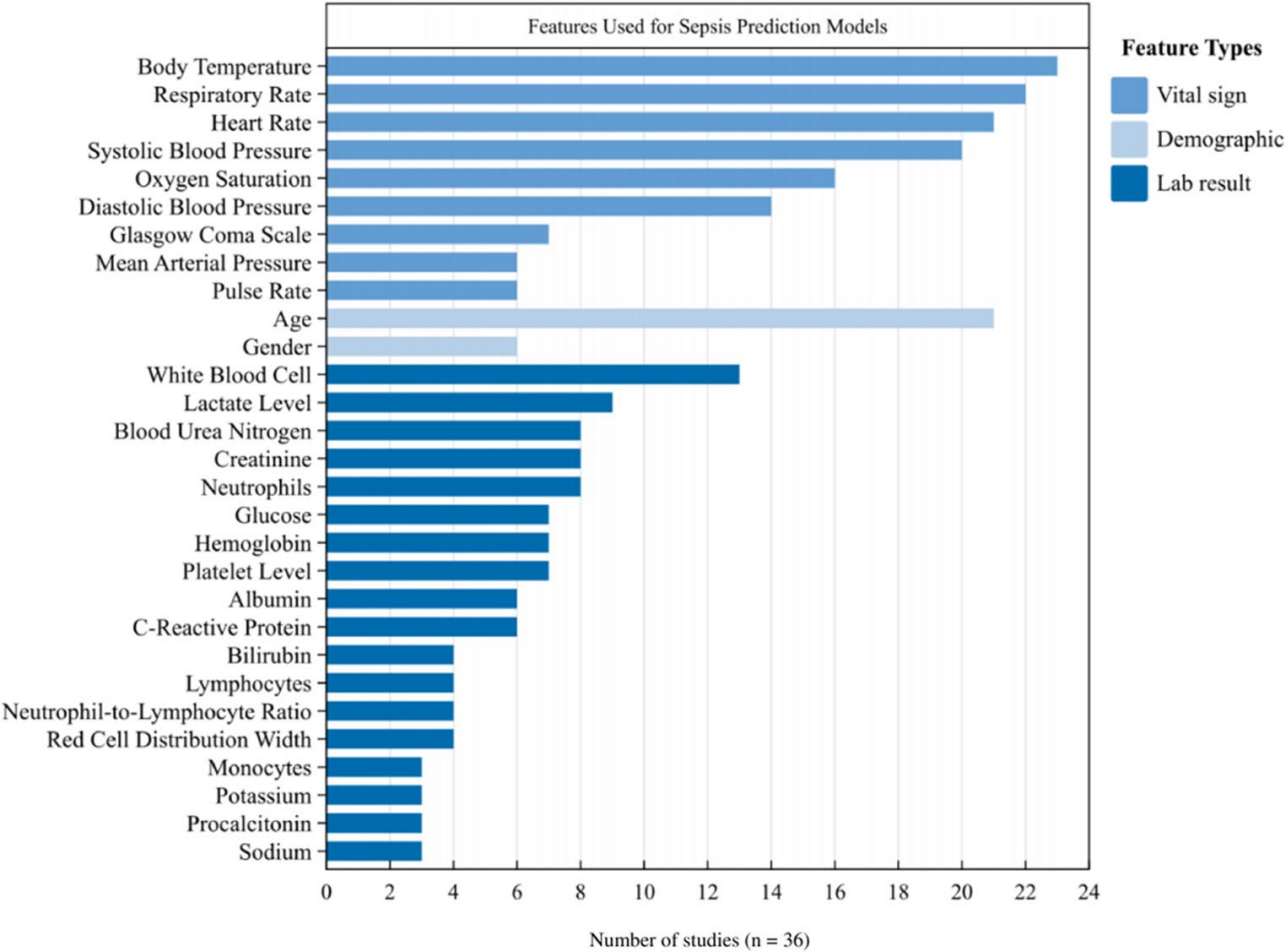
Common features used for sepsis prediction models

The 29 most frequently used predictive features were categorized as vital signs, demographics, and laboratory results (Fig. 4). The top five commonly reported features across studies (> 50%) were body temperature, respiratory rate, heart rate, systolic blood pressure, and age.

###  Quality of Reporting

**Fig. 5:**
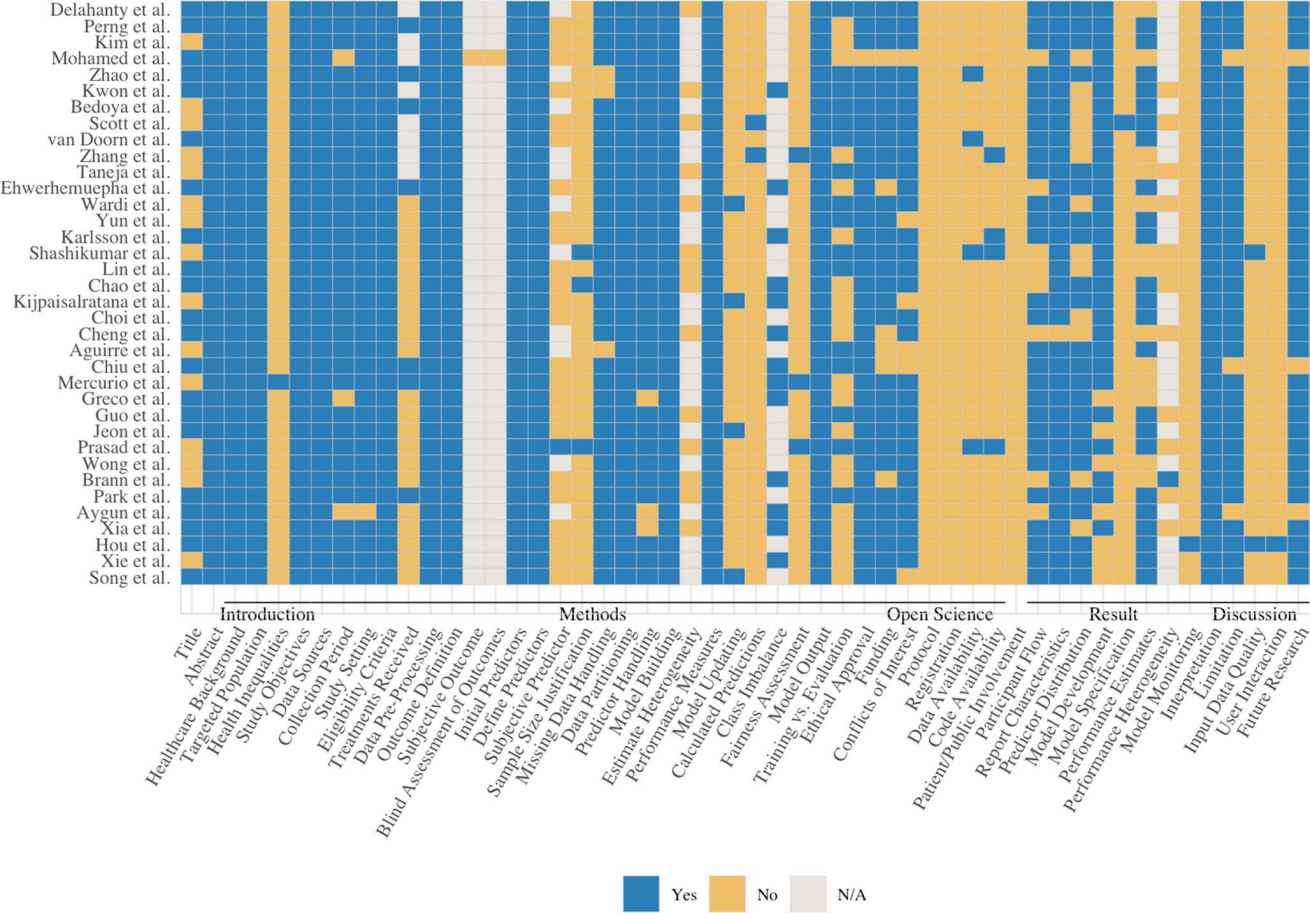
Quality assessment results of included studies

Across 36 included studies appraised with TRIPOD + AI (Fig. 5), basic descriptive and methodological items were well-reported; whereas major deficiencies remained in open-science practices and in the level of result completeness needed for external validation (full item-level ratings were provided in Table S8)

#### Title, Abstract, Introduction

The assessment criteria for abstract, healthcare background, targeted population, and study objectives were fulfilled in all studies. In comparison, explicit identification of prediction model development in the title was not frequently presented (*n* = 21, 58%), and health-inequality considerations across sociodemographic groups were rarely recognized (*n* = 1, 3%).

#### Methods

Information on cohort definition and data provenance was reported in nearly all reports, including data sources, study dates and settings, eligibility criteria, outcome definitions, and preprocessing pipelines. Predictors were typically defined with missingness handling and data partitioning, and model development with performance measures and outputs was presented. Ethical approval was also commonly reported. By contrast, several safeguards and reproducibility enablers were inconsistently reported. Training-evaluation differences were described in around half of studies (*n* = 16, 44%); details of treatments received that could influence sepsis prediction outcomes were reported in only 7 studies (19%); model updating, fairness assessment, sample-size justification and calculated prediction were seldom summarized (no more than 11%).

#### Open Science and Patient/Public Involvement

Open-science fidelity was limited. Protocols, registrations, and patient/public involvement were unavailable, and data/code availability was uncommon (each *n* = 4, 11%). However, funding and conflicts of interest were more often disclosed (each *n* = 29, 83%).

#### Results

Most studies shared similar trends in their resulting outputs, and participant flow and analytical cohort were clearly presented. The dataset characteristics (*n* = 35, 97%) were frequently outlined, participant flows, predictor distributions, model development steps, and overall performances were reported in over 50%. As a result, while headline performance was often visible, transparency needed for recalculation, audit, or external validation remained limited, full model specification was rarely sufficient for independent reconstruction, and forward-looking plans for monitoring in use were almost never provided (each *n* = 1, 3%).

#### Discussion

The interpretation of findings was clarified across included studies (*n* = 36, 100%), with limitations and future directions considered (*n* = 33, 92%). However, practice-facing details needed for ED adoption were hardly addressed. Approaches for managing input-data quality at the point of use were rarely specified (*n* = 2, 6%), and practical requirements for end-user interaction were almost never described (*n* = 1, 3%). Therefore, operational readiness and guidance for safe deployment remain underdeveloped despite thoughtful reflections.

###  Meta-Analyses

#### Pooled Prediction Performance

The random effects meta-analysis was used to integrate performance metrics from 98 AI models across 36 included studies given the observed high heterogeneity ($\:{I}^{2}$ > 50%). The pooled AUROC for algorithmic sepsis prediction in EDs was 0.87 (95% CI: 0.86–0.88, $\:{I}^{2}$ = 99.94%). Corresponding pooled estimates of accuracy, sensitivity, and specificity were respectively 0.81 (95% CI: 0.79–0.83, $\:{I}^{2}$ = 99.92%), 0.77 (95% CI: 0.74–0.81, $\:{I}^{2}$ = 99.98%), and 0.85 (95% CI: 0.83–0.87, $\:{I}^{2}$ = 99.99%).

**Fig. 6:**
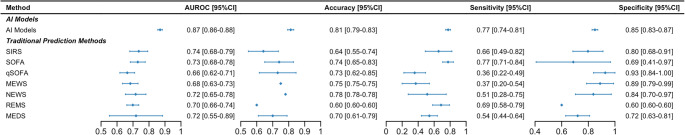
Comparison of pooled performance between AI-based models and traditional rule-based scores. Multivariate Imputation by Chained Equations

By comparison, reported traditional rule-based methods (SIRS, SOFA, qSOFA, MEWS, NEWS, REMS, MEDS) showed lower pooled AUROCs (0.66–0.74), pooled accuracy (0.60–0.78), and pooled sensitivity (0.36–0.77) in 23 included studies (Fig. 6 and Table S9). Although the specificity of AI models was comparable with some traditional tools (e.g., qSOFA at 0.93, MEWS at 0.89, and NEWS at 0.84), it remained consistently high across evaluations. Pooled estimates for conventional scores were based on the subset of studies reporting each metric, and the AI-score comparisons should be interpreted with this in mind.

#### Subgroup Analysis

**Fig. 7:**
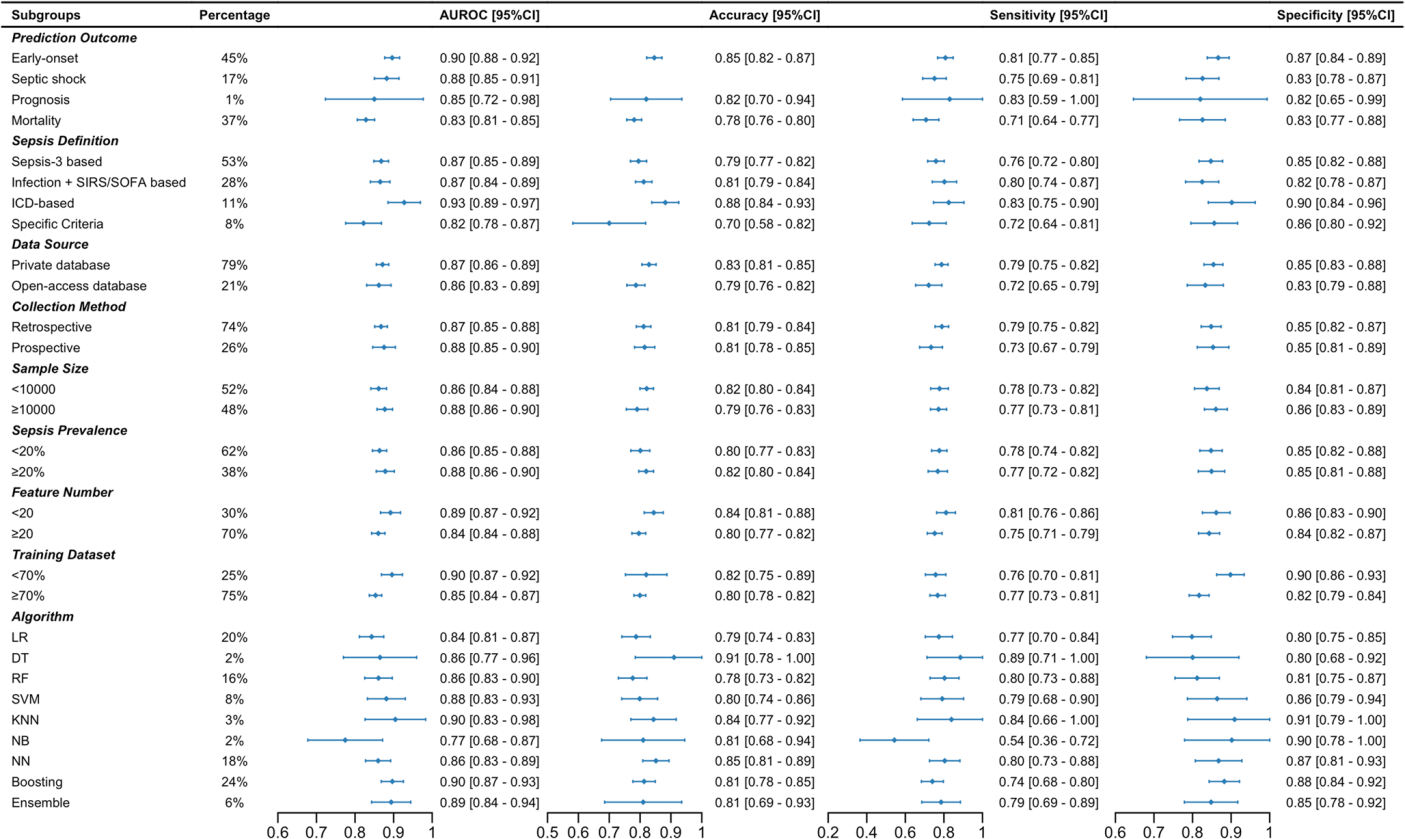
Pooled performance measures in subgroups. Prediction outcome significantly moderated AUROC (QM(3)=21.18, p < 0.001; mainly mortality vs early-onset). The prognosis subgroup was based on a single study/model (k=1) and is presented descriptively. Accuracy was not reported by any septic shock prediction studies and is shown as not available

In subgroup analyses (Fig. 7), higher pooled AUROC values were observed in models targeting early-onset sepsis (0.90; 95%CI: 0.88–0.92), using International Classification of Diseases (ICD) based definitions (0.93; 95% CI: 0.89–0.97), private medical databases (0.87; 95% CI: 0.86–0.89), prospective data collection (0.88; 95% CI: 0.85–0.90), larger samples (≥ 10,000) (0.88; 95% CI: 0.86–0.90) with higher prevalence (≥ 20%) (0.88; 95% CI: 0.86–0.90), fewer features (< 20) (0.89; 95% CI: 0.87–0.92), and smaller training proportions (< 70%) (0.90; 95% CI: 0.87–0.92). Among the algorithms covered, boosting-based models exhibited the best pooled AUROC (0.90; 95% CI: 0.87–0.93), followed by KNN (0.90; 95% CI: 0.83–0.98), ensemble methods (0.89; 95% CI: 0.84–0.94), and SVM (0.88; 95% CI: 0.83–0.93).

#### Modeling Methodological Predictors

**Fig. 8:**
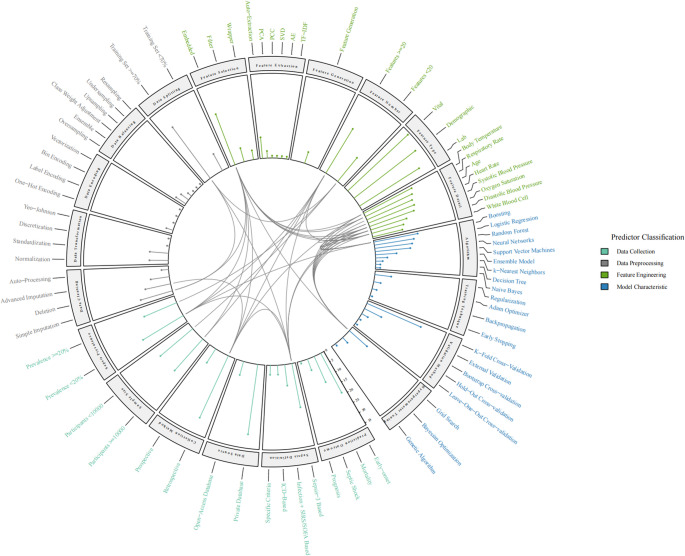
Circle plot of predictor classifications, frequency distributions, and significant positive interactions among high-frequency predictors in AI-based sepsis prediction models. Simple imputation: missing values were replaced using fixed or rule-based methods, such as mean/median substitution or carry-forward filling. Advanced imputation: missing values were estimated using model-based approaches, such as Multivariate Imputation by Chained Equations (MICE) or random forest–based imputation

80 identified predictors are visualized in a circular plot (Fig. 8). Beyond descriptive co-occurrence and frequency counts, pairwise statistically significant positive interactions among predictors that appeared in at least five studies were harmonized to prespecified classifications (data collection, data preprocessing, feature engineering, and model characteristics).

Explicit reporting of these predictors revealed model development frameworks and facilitated transparency and comparability across studies. As the foundation for subsequent modeling, specific aspects of data collection were subdivided into prediction outcomes, sepsis definitions, data sources, collection methods, sample sizes and prevalence; data preprocessing covered cleaning for outliers and missing values, data transformation, encoding, balancing and splitting; feature engineering involved feature selection, extraction, generation [30], counts and types; and model characteristics included algorithm selection, hyperparameter tuning, and a series of training and validation techniques.

Interaction motivated the later regression work by showing recurring methodological combinations across studies. These co-occurrence patterns were used to reduce dimensionality and narrow down candidate predictors, rather than to support pairwise performance claims. Low-prevalence or imbalanced settings were repeatedly linked to strategies that strengthened minority-class signals, such as embedded feature selection and boosting-type learners. Common physiological markers (Fig. 4), including heart rate, blood pressure, and oxygen saturation, were frequently involved in positive interactions with feature selection, hyperparameter tuning, and boosting algorithms. Data scale and splitting design also interacted with feature-engineering decisions, with larger cohorts generally well-suited for embedded feature selection, while smaller feature groups often required higher training sets for full learning.

#### Three-Step Regression Analysis

Meta-regression was used to investigate potential sources of heterogeneity and to identify which study-level variables were associated with model predictiveness, while recognizing that these comparisons reflected methodological combinations rather than isolated causal effects. To ensure data comparability across studies, 40 of the 80 high-frequency predictors (Fig. 8) were selected for regression analyses. Considering the substantial heterogeneity, mixed-effects meta-regressions on the logit scale of AUROC were undertaken (Table 1).

**Table 1:** Univariate and multivariate outcomes of meta-regressions

Variables	Univariate Model			Multivariate Model			Significant Variable Model		
	β	*p*	95% CI	β	*p*	95% CI	β	*p*	95% CI
Prediction Outcome: Early-onset	0.269	0.701	[−1.102, 1.640]	−0.102	0.893	[−1.591, 1.387]			
Prediction Outcome: Septic shock	0.066	0.930	[−1.413, 1.545]	0.198	0.582	[−0.508, 0.905]			
Prediction Outcome: Mortality	0.403	0.564	[−0.968, 1.774]	0.033	0.965	[−1.456, 1.522]			
Sepsis definition: Sepsis-3 based	−0.140	0.521	[−0.567, 0.287]	−0.677	0.031*	[−1.293, −0.062]	−0.042	0.839	[−0.463, 0.380]
Sepsis definition: Infection + SIRS/SOFA	−0.228	0.362	[−0.718, 0.262]	−0.605	0.086	[−1.294, 0.085]			
Data Source: Private database	−0.009	0.975	[−0.582, 0.564]	−0.008	0.979	[−0.622, 0.606]			
Collection Method: Retrospective	−0.041	0.883	[−0.582, 0.501]	−0.532	0.114	[−1.191, 0.127]			
Sample Size ≥ 10000	0.355	0.092	[−0.057, 0.767]	0.820	0.004*	[0.263, 1.378]	0.449	0.011	[0.116, 0.782]
Sepsis Prevalence	0.285	0.670	[−1.027, 1.597]	1.642	0.051	[−0.006, 3.289]			
Data cleaning: Simple imputation	0.091	0.685	[−0.348, 0.530]	−0.297	0.260	[−0.815, 0.220]			
Data cleaning: Advanced imputation	−0.361	0.158	[−0.864, 0.141]	−0.138	0.609	[−0.669, 0.392]			
Data cleaning: Deletion	0.387	< 0.001*	[0.373, 0.401]	0.387	< 0.001*	[0.373, 0.401]	0.409	0.003*	[0.286, 0.531]
Data Transformation: Normalization	−0.524	0.034*	[−1.010, −0.039]	−0.428	0.100	[−0.938, 0.082]	−0.234	0.393	[−0.841, 0.372]
Data Transformation: Standardization	0.221	0.424	[−0.320, 0.762]	0.653	0.026*	[0.079, 1.228]	0.318	0.199	[−0.201, 0.837]
Data Balancing: Oversampling	0.086	0.772	[−0.494, 0.666]	0.497	0.080	[−0.060, 1.055]			
Data Splitting: Training Set ≥ 70%	−1.641	0.125	[−3.739, 0.458]	−0.738	0.015*	[−1.331, −0.146]	−0.284	0.371	[−0.950, 0.381]
Feature Selection: Filter	−0.421	0.141	[−0.982, 0.140]	−0.651	0.035*	[−1.257, −0.045]	−0.182	0.381	[−0.639, 0.275]
Feature Selection: Embedded	−0.213	0.344	[−0.653, 0.228]	−0.238	0.311	[−0.697, 0.222]			
Feature Extraction: Auto-extraction	0.061	< 0.001*	[0.052, 0.070]	0.061	< 0.001*	[0.052, 0.070]	0.172	0.522	[−1.186, 1.530]
Feature Number	−0.006	0.125	[−0.014, 0.002]	−0.006	0.157	[−0.014, 0.002]			
Feature Type: Demographic	−0.886	0.019*	[−1.626, −0.146]	−1.000	0.042*	[−1.963, −0.036]	−0.995	0.035*	[−1.861, −0.128]
Feature Type: Vital	−0.529	0.421	[−1.818, 0.760]	0.862	0.263	[−0.646, 2.370]			
Feature Type: Lab	0.085	0.771	[−0.491, 0.662]	0.359	0.263	[−0.269, 0.986]			
Feature: Body Temperature	0.047	0.843	[−0.418, 0.512]	0.101	0.752	[−0.523, 0.724]			
Feature: Respiratory Rate	−0.054	0.813	[−0.509, 0.400]	0.143	0.654	[−0.483, 0.770]			
Feature: Age	−0.424	0.051	[−0.849, 0.002]	−0.487	0.092	[−1.054, 0.080]			
Feature: Heart Rate	−0.102	0.649	[−0.540, 0.337]	−0.098	0.715	[−0.626, 0.429]			
Feature: Systolic Blood Pressure	−0.074	0.743	[−0.514, 0.367]	0.107	0.784	[−0.656, 0.869]			
Feature: Oxygen Saturation	−0.023	0.918	[−0.455, 0.410]	0.034	0.889	[−0.447, 0.516]			
Feature: Diastolic Blood Pressure	−0.096	0.664	[−0.530, 0.338]	−0.122	0.723	[−0.797, 0.553]			
Feature: White Blood Cell	0.271	0.216	[−0.159, 0.701]	0.182	0.464	[−0.305, 0.669]			
Algorithm: Logistic Regression	0.128	< 0.001*	[0.118, 0.139]	0.112	< 0.001*	[0.101, 0.123]	0.138	0.624	[−1.317, 1.593]
Algorithm: Random Forest	0.204	< 0.001*	[0.194, 0.215]	0.213	< 0.001*	[0.203, 0.224]	0.261	0.379	[−0.928, 1.450]
Algorithm: Support Vector Machines	0.335	< 0.001*	[0.316, 0.355]	0.336	< 0.001*	[0.317, 0.356]	0.369	0.147	[−0.283, 1.021]
Algorithm: Boosting	0.180	< 0.001*	[0.168, 0.192]	0.188	< 0.001*	[0.177, 0.200]	0.208	0.430	[−0.990, 1.405]
Algorithm: Neural Networks	0.261	< 0.001*	[0.247, 0.274]	0.276	< 0.001*	[0.262, 0.289]	0.045	0.523	[−0.327, 0.416]
Regularization	0.191	0.491	[−0.352, 0.733]	0.226	0.409	[−0.310, 0.761]			
K-fold Cross-validation	0.265	0.257	[−0.193, 0.723]	0.247	0.300	[−0.221, 0.716]			
Hyperparameter Tuning: Grid search	0.073	< 0.001*	[0.049, 0.097]	0.186	< 0.001*	[0.160, 0.212]	0.163	0.131	[−0.178, 0.504]
External Validation	−0.166	0.526	[−0.678, 0.347]	−0.026	0.922	[−0.545, 0.493]			

Values are reported to three decimal places; * denotes statistically significant correlations with AUROC (p < 0.05). Coefficients are contrasts relative to the reference on the logit-AUROC scale; sign changes when the reference changes.

Independent associations were first explored in univariate regressions. Higher AUROC was observed in studies reporting deletion for data cleaning, automated feature extraction, grid-search for hyperparameter tuning, and the use of mainstream algorithms (LR, RF, SVM, NN, and boosting). In contrast, normalization for data transformation and inclusion of demographic features were associated with lower AUROCs in unadjusted models.

Multivariate meta-regressions were fitted within prespecified methodological blocks (data collection, data preprocessing, feature engineering with types, and model characteristics; see Fig. 8) to limit collinearity and reduce overfitting. Several univariate variables attenuated after mutual adjustment, while the overall direction of associations remained consistent. Higher AUROC was basically associated with larger sample size (≥ 10,000), deletion-based and standardization-based data processing, feature auto-extraction, and grid-search tuning. Sepsis-3 definition, filter-based feature selection, demographic inclusion, and larger training allocation (≥ 70%) were linked to lower performance.

An integrated model was then used to estimate the joint effects of significant variables identified from univariate and multivariate screens. The integrated model showed an improvement in fit (AICc reduced by 5,827.71) and a reduction in between-study variance ($\:{\tau\:}^{2}$ from 0.38 to 0.25; pseudo-$\:{R}^{2}$ = 33.83%) compared to the baseline random-effects model, indicating that these study-level variables explained a meaningful proportion of between-study heterogeneity. Statistically supported associations were retained for sample size, deletion-based data cleaning, and use of demographic features. Although algorithm families exhibited significant positive associations in the earlier two-step regressions, their contrasts under the integrated model remained directionally positive but were estimated with uncertainty.

#### Publication Bias and Sensitivity Analysis

The result of Egger’s test indicated no significant publication bias in this research ($\:t$ = 0.04, *P*-value = 0.96 > 0.05), confirmed by a symmetrical funnel plot (Fig S1). Sensitivity analysis demonstrated stable pooled estimates, almost unaffected by individual studies, affirming the robustness of our findings.

##  Discussion

###  Principal Findings

This systematic review and meta-analysis provides a comprehensive evaluation of recent AI-based sepsis prediction studies in emergency contexts, including 36 studies with 98 models from the past five years. Consistent with the prior research [11, 29, 30, 73], this work suggests that AI-assisted models achieved higher performance than widely-adopted traditional clinical screening tools. Specifically, the pooled AUROC for AI models (0.87) exceeded that of traditional methods (0.66–0.74).

Conventional rule-based scores are typically developed for broad assessment of infection-related illness rather than for explicit sepsis prediction [74]. By comparison, AI-based models can incorporate large-scale, multi-source, and high-dimensional clinical data, which may allow them to capture subtle and dynamic physiological changes that threshold-based criteria do not fully detect [12]. However, the significant heterogeneity observed in this review, consistent with previous meta-analyses [11, 25, 34], necessitating deeper consideration of underlying methodological predictors, as well as a cautious explanation of pooled results. Sepsis-prediction model development involves complex processes, and predictors for data preparation and model characterization undeniably affect performance to varying degrees.

###  Methodological Interpretation

Compared with existing reviews that mainly summarized predictive performance [32, 75], this study expands the scope through subgroup and regression analyses to identify methodological sources of heterogeneity and inform both model development and implementation. Some methodological choices may still be useful, but their independent contribution becomes harder to isolate when accounting for cohort scale and data preprocessing variables. Meanwhile, the negative associations also have interpretive value in that a lower AUROC does not necessarily imply poorer methodological quality [52, 66].

#### Outcome Definition and Prediction Task

Subgroup analyses suggested higher pooled discrimination under ICD-based labels, whereas regression analyses showed a negative association for Sepsis-3-based definitions. These findings indicate that sepsis definitions are interpreted as different prediction targets rather than interchangeable labels. ICD-based definitions are more established in routine administrative records, corresponding more closely to later clinician-confirmed states. In comparison, Sepsis-3 is closer to earlier physiologic identification, and lower performance under it reflects a stricter prediction task, which cannot be directly explained as evidence of inferior modeling.

#### Cohort and Data Context

At the cohort and data level, higher performance tended to be reported in studies using private databases, prospective collection, larger samples, and higher prevalence. Regression findings further examined that sample size remained one of the most reproducible independent variables in the integrated model. Larger sample sizes potentially reduce estimation instability and better represent heterogeneous ED presentations. These characteristics are more consistent with differences in data fidelity, cohort scale, and class balance than with any single optimal design rule [23, 73].

#### Data Preprocessing and Feature Representation

Findings related to data preprocessing and feature representation indicated that model performance depended not only on how much information was available, but also on how that information was cleaned and structured. Subgroups suggested that models using fewer clinical features could perform comparably to those using more high-dimensional sets [30, 43]. In regressions, higher AUROCs were associated with deletion-based cleaning, standardized transformation, and automated feature extraction, while larger training allocation, demographic inclusion, and filter-based selection were linked to lower performance.

The advantage of smaller training-to-validation ratios can be understood as the presence of broader validation cohorts to produce more reliable estimates. It is not a universally preferable split or an inherent benefit of allocating less data to model development [76, 77]. Similarly, the inverse correlation for filter-based feature selection may involve missing interaction-driven or time-dependent information common in ED sepsis progression [30]. For preprocessing-related variables, standardization showed a stronger association after adjustments, which is reasonable when features measured on different scales are jointly analyzed [78]. In contrast, the earlier univariate pattern for normalization weakened in the block-wise multivariate model, implying that it is largely related to the modeling context in which normalization is applied than to an adverse effect of normalization itself.

In the integrated model, deletion-based cleaning and demographic features remained statistically supported. The positive signal for deletion may reflect the efficiency and simplicity compared to inappropriate imputation methods. Simultaneously, consistent with previous findings, when dealing with larger sample sizes, lower baseline missing rates, and simpler cohort structures, deletion methods can ensure high-quality training data free from artificial value interference. The negative relationship for demographics does not represent that such variables are clinically unimportant. It reveals that static demographics add limited short-term discrimination once dynamic physiological information (e.g., vital signs and laboratory data) is encoded, while still remaining relevant for patient calibration, subgroup reporting, and fairness assessment [43, 48, 52, 68].

#### Model-Building Choices

The independent contribution of methods used in model construction was less stable than that of cohort and preprocessing variables. Subgroup analyses examined relatively strong pooled AUROC for boosting-based models, and early regression phases also showed positive correlations for several commonly used algorithms and grid-search tuning. Nevertheless, these contrasts attenuated in the integrated model, suggesting that part of their advantages may stem from context-dependent design, data, and feature-engineering decisions.

###  Methodological Synthesis

These findings indicate that heterogeneity in ED sepsis prediction studies is shaped by interactions among target definitions, cohort structures, preprocessing strategies, feature representations, and model-development choices. Methodological benchmarks should therefore prioritize clear specification of prediction targets, index time, prediction windows; representative and temporally consistent cohorts [42, 61, 66]; transparent handling of missingness, outliers, and feature scaling [77, 79, 80]; and systematic tunings that enhance model fitting and validation strategies that preserve adequate evaluation scopes.

The considerations are reinforced by TRIPOD + AI appraisal, which clarified why many studies lacked details required for independent reconstruction and clinical translation, including open-science practices, reproducible model specification, operating thresholds, calibration outputs, and external/temporal validation. Consequently, methodological benchmarks should be framed as a pathway to improve cross-study comparability, as well as a prerequisite for reconstruction-level standardization and deployment-oriented evaluation.

### From Theory to Practice: Implementation Benchmarks

#### Standardization and Clinical Impacts

The implication of establishing benchmarks extends beyond model development to the credible evaluation and effective deployment of sepsis prediction tools in routine emergency care. Despite demonstrating obvious numerical strengths in predictive accuracy, limited external validations and actual implementations revealed in TRIPOD + AI highlight major challenges to adoption within emergency medical services. It is widely acknowledged in current studies that establishing standardized methods is a foundational step towards transparency [30, 74, 81]. Furthermore, this also encourages emergency professionals to evaluate and incorporate AI-based systems into clinical workflows with clearer operational expectations [5–9, 80].

Standardized methodologies may also improve clinical accountability through systematic monitoring of model outcomes [82], including the effectiveness of implementing clinical interventions based on predictive results. Clear standards allow tracking of unintended consequences, such as alert fatigue or inefficient resource use [20, 83, 84]. Explicit reporting requirements can further support regulatory oversight and compliance with healthcare quality standards, ensuring AI-driven clinical decision support systems uphold patient safety and ethical responsibility in practice.

#### Implementation Strategies

The analysis supports an implementation-focused approach instead of treating model performance as the ultimate goal. One of the most critical practical barriers is to translate model outputs into reproducible clinical pathways. Reliability, interpretability and actionability should be emphasized during deployment. Sepsis prediction models should be embedded at decision points with prespecified use cases, as reported performance is highly sensitive to target definitions and evaluation designs. It is important to state the prediction window ranges, select operational thresholds that align with local system capacity, and provide calibration results so that risk scores have consistent clinical meaning [76, 85].

Sustainable benefits also depend on whether model performance remains stable under routine ED data constraints. Because the more reproducible performance differences in this review were linked to cohort scale and data processing, implementation should prioritize reliable input streams based on routinely available dynamic signals, together with transparent rules for missing, abnormal, or delayed measurements. Ongoing monitoring should assess whether predicted risks continue to match observed event rates and whether alert burden is acceptable for ED workflow [44]. In addition, responses to performance drifts should be defined in advance and managed through recalibration and reassessment, which has opportunities to avoid repeated algorithm modifications.

In theory, future work needs to explore why strategically simplified models may at time outperform more complex approaches that blindly increase feature sizes or training scales. Direct comparisons between parsimonious and high-dimensional models are also required to identify whether the advantages of simpler designs represent reduced overfitting, enhanced calibration, or effective transportability across settings. Research should focus on temporal effects, feature interactions, and target definitions influencing model behaviors, particularly across different sepsis criteria, workflow stages, and patient subgroups. Moreover, fairness and stability should be evaluated more systematically to determine if predictive performance is maintained across clinically and demographically diverse populations [25, 50].

###  Future Directions

In practice, further studies should plan prospective external validations and multicentre implementation-oriented research to validate whether performance can be sustained outside of single-site retrospective datasets. Evaluation should move beyond AUROC measures to include calibration accuracy, threshold-based clinical operability, workflow integration, resource use [20, 30], and patient-centred outcomes. To improve real-world readiness, models should be developed under ethical and governance requirements that specify intended use, support transparent reporting, and enable post-deployment monitoring [86]. Greater emphasis on reproducibility, transportability, and clinically embedded evaluation will be important for strengthening AI-based sepsis prediction from promising technical performance to a more accountable emergency care application.

##  Limitations

This review inevitably has several limitations. The heterogeneity across included studies remained due to differences ranging from data collection to modeling phases, despite attempts to dissect the algorithm development behind the outstanding performance. Evidence certainty assessment based on prediction outcome categories using GRADEpro GDT shows overall low certainty, with a very low level for prognostic assessment due to methodological limitations and heterogeneity (Table S10). As a result, the pooled estimates should be interpreted with caution. Incomplete reporting of accuracy, sensitivity and specificity reduces the precision of secondary pooled analyses, whereas the representativeness and appropriateness of AUROC as a primary result metric remain controversial.

##  Conclusion

This systematic review and meta-analysis indicates that AI-based models achieve higher pooled predictive performance for sepsis compared with traditional screening systems in emergency departments. However, substantial methodological heterogeneity and limited external validation constrain generalizability. The standardized methodological and reporting benchmarks have the potential to improve cross-study reproducibility, comparability, and readiness towards clinical implementation.

## Supplementary Information

Below is the link to the electronic supplementary material.

Supplementary File 1 (DOCX 206 KB)

## Data Availability

No datasets were generated or analysed during the current study.
