# Supplementary material for: Evaluating Artificial Intelligence for Sepsis Prediction in Emergency Departments: A Systematic Review and Meta Analysis
Source: J Med Syst. 2026 Apr 13;50(1):49. doi: 10.1007/s10916-026-02376-3 (PMC13076368; doi:10.1007/s10916-026-02376-3)
Supplement: Supplementary file 1 — (DOCX 206 KB) [file 10916_2026_2376_MOESM1_ESM.docx]

### Supplementary material

Title:

Evaluating Artificial Intelligence for Sepsis Prediction in Emergency Departments: A Systematic Review and Meta Analysis

Journal:

Journal of Medical Systems

Authors:

Yinan Zhang, Tim Kirchler, Audrey P Wang (corresponding author)

Affiliation:

The University of Sydney

E-mail:

audrey.wang1@sydney.edu.au

##### 1 Search Strategy Methodology

###### 1.1 Search Terms

1.1.1 Synonyms or alternative search terms

(1) Sepsis: "Septic*", "Systemic Inflammatory Response Syndrome", "SIRS", "SOFA", "qSOFA", "Sequential Organ Failure Assessment"

1. AI: "Artificial Intelligence", "Computational Intelligence", "Machine Intelligence", "Machine Learning", "Deep Learning", "Neural Network", "Natural Language Processing", "Decision tree*", "Random Forest", "Support Vector Machine", "Gradient Boosting", "Naïve Bayes", "Adaboosting", "Regression"
2. Emergency Department: "Emergency Department*", "Emergency Room*", "Emergency Service*", "Emergency Ward*", "Emergency Unit*", "Emergency Hospital*", "Emergency Medical Service*", "Emergency Patient*", "Emergency Admiss*", "Emergency Admit*", "Accident and Emergency", "Emergenc*"
3. Prediction: "Predict*", "Diagnos*", "Prognos*", "Detect*", "Identif*"

1.1.2 Subject Headings: (MeSH & Emtree 2024)

MeSH: for PubMed and MEDLINE

(1) Sepsis: "Sepsis", "Systemic Inflammatory Response Syndrome"

(2) AI: "Algorithms", "Artificial Intelligence", "Machine Learning", "Natural Language Processing", "Neural Networks, Computer"

(3) Emergency Department: "Emergency Medical Services", "Emergency Medicine"

(4) Prediction: "Diagnosis", "Prognosis"

Emtree: for Embase

1. Sepsis: "Sepsis", "Systemic Inflammatory Response Syndrome"
2. AI: "Artificial Intelligence", "Machine Learning", "Natural Language Processing", "Decision Tree", "Big Data"
3. Emergency Department: ""Emergency Health Service", "Emergency Medicine", "Emergency Ward", "Hospital Emergency Service"
4. Prediction: "Prediction", "Predictive Model", "Diagnosis", "Prognosis"

1.1.3 Concept Table

**Table S1. Concept table.**

| **Concept** | **Synonyms / Alternative Terms** | **MeSH Subject Headings** | **Emtree Subject Headings** | **Adjustment Plan** |
| --- | --- | --- | --- | --- |
| Sepsis | "Septic*", "Systemic Inflammatory Response Syndrome", "SIRS", "SOFA", "qSOFA", "Sequential Organ Failure Assessment" | "Sepsis", "Systemic Inflammatory Response Syndrome" | "Sepsis", "Systemic Inflammatory Response Syndrome" | To consider sepcific types of sepsis if necessary |
| AI | "Artificial Intelligence", "Computational Intelligence", "Machine Intelligence", "Machine Learning", "Deep Learning", "Neural Network", "Natural Language Processing", "Decision tree*", "Random Forest", "Support Vector Machine", "Gradient Boosting", "Naïve Bayes", "Adaboosting", "Regression" | "Algorithms", "Artificial Intelligence", "Machine Learning", "Natural Language Processing", "Neural Networks, Computer" | "Artificial Intelligence", "Machine Learning", "Natural Language Processing", "Decision Tree", "Big Data" | To contain narrower terms of AI methods like "Machine Learning" and its algorithms |
| Emergency Department | "Emergency Department*", "Emergency Room*", "Emergency Service*", "Emergency Ward*", "Emergency Unit*", "Emergency Hospital*", "Emergency Medical Service*", "Emergency Patient*", "Emergency Admiss*", "Emergency Admit*", "Accident and Emergency", "Emergenc*" | "Emergency Medical Services", "Emergency Medicine" | "Emergency Health Service", "Emergency Medicine", "Emergency Ward", "Hospital Emergency Service" | To consider broader terms like "Acute Care" if there are not enough search results |
| Prediction | "Predict*", "Diagnos*", "Prognos*", "Detect*", "Identif*" | "Diagnosis", "Prognosis" | "Prediction", "Predictive Model", "Diagnosis", "Prognosis" | To make search results more targeted but include terms with similar meanings |

This table organizes our search strategy for concepts, synonyms, subject headings and plans for combination. The adjustment plan section includes guidance on how these terms will be interconnected and relevant to the research.

###### 1.2 Search Query Strings (Nov 8, 2024)

1.2.1 Search in PubMed

1. Sepsis: 284,396

Start with the MeSH term for sepsis and include some relevant keywords to capture studies.

( Sepsis[MeSH] OR "Systemic Inflammatory Response Syndrome"[MeSH] OR Sepsis[Title/Abstract] OR Septic*[Title/Abstract] OR "Systemic Inflammatory Response Syndrome"[Title/Abstract] OR SIRS[Title/Abstract] OR SOFA[Title/Abstract] OR qSOFA[Title/Abstract] OR "Sequential Organ Failure Assessment"[Title/Abstract] )

1. AI: 1,804,729

Contains specific technologies and synonyms that are commonly used in AI methods, as "Artificial Intelligence" is a broad term.

( Algorithms[MeSH] OR "Artificial Intelligence"[MeSH] OR "Machine Learning"[MeSH] OR "Natural Language Processing"[MeSH] OR "Neural Networks, Computer"[MeSH] OR "Artificial Intelligence"[Title/Abstract] OR AI[Title/Abstract] OR "Computational Intelligence"[Title/Abstract] OR "Machine Intelligence"[Title/Abstract] OR "Machine Learning"[Title/Abstract] OR "Deep Learning"[Title/Abstract] OR "Neural Network"[Title/Abstract] OR "Natural Language Processing"[Title/Abstract] OR "Decision tree*"[Title/Abstract] OR "Random Forest"[Title/Abstract] OR "Support Vector Machine"[Title/Abstract] OR "Gradient Boosting"[Title/Abstract] OR "Naïve Bayes"[Title/Abstract] OR Adaboosting[Title/Abstract] OR Regression[Title/Abstract] )

1. Emergency Department: 624,183

Use the MeSH terms and common synonyms and abbreviations for a search.

( "Emergency Medical Services"[MeSH] OR "Emergency Medicine"[MeSH] OR "Emergency Department*"[Title/Abstract] OR "Emergency Room*"[Title/Abstract] OR "Emergency Service*"[Title/Abstract] OR "Emergency Ward*"[Title/Abstract] OR "Emergency Unit*"[Title/Abstract] OR "Emergency Hospital*"[Title/Abstract] OR "Emergency Medical Service*"[Title/Abstract] OR "Emergency Patient*"[Title/Abstract] OR "Emergency Admiss*"[Title/Abstract] OR "Emergency Admit*"[Title/Abstract] OR "Accident and Emergency"[Title/Abstract] OR Emergenc*[Title/Abstract] )

1. Prediction: 7,321,841

Adopt the concept and its MeSH terms, synonyms and alternative words for the retrieval.

( Diagnosis[MeSH] OR Prognosis[MeSH] OR Predict*[Title/Abstract] OR Diagnos*[Title/Abstract] Prognos*[Title/Abstract] OR Detect*[Title/Abstract] OR Identif*[Title/Abstract] )

1. Combining the concepts and filtering conditions: 667

We have already defined the search terms for each concept and combined these concepts with the Boolean operator (AND) to ensure that the retrieved studies are relevant to key aspects of our research question, we also set the time range from 2019 to 2024 to ensure the latest research results.

1.2.2 Search in Scopus

1. Sepsis: 430,599

TITLE-ABS-KEY ( sepsis OR septic* OR "Systemic Inflammatory Response Syndrome" OR sirs OR sofa OR qsofa OR "Sequential Organ Failure Assessment" )

2. AI: 4,628,958

TITLE-ABS-KEY ( "Artificial Intelligence" OR ai OR "Computational Intelligence" OR "Machine Intelligence" OR "Machine Learning" OR "Deep Learning" OR "Neural Network" OR "Natural Language Processing" OR "Decision Tree*" OR "Random Forest" OR "Support Vector Machine" OR "Gradient Boosting" OR "Bayes" OR "Adaboosting" OR "Regression" )

3. Emergency Department: 1,245,655

TITLE-ABS-KEY ( "Emergency Department*" OR "Emergency Room*" OR "Emergency Service*" OR "Emergency Ward*" OR "Emergency Unit*" OR "Emergency Hospital*" OR "Emergency Medical Service*" OR "Emergency Patient*" OR "Emergency Admiss*" OR "Emergency Admit*" OR "Accident and Emergency" OR emergenc* )

4. Prediction: 23,654,106

TITLE-ABS-KEY ( predict* OR diagnos* OR prognos* OR detect* OR identif* )

5. Combination with filtering conditions: 1,508

1.2.3 Search in Web of Science

1. Sepsis: 284,755

TS=(sepsis OR septic* OR "Systemic Inflammatory Response Syndrome" OR sirs OR sofa OR qsofa OR "Sequential Organ Failure Assessment") OR TI=(sepsis OR septic* OR "Systemic Inflammatory Response Syndrome" OR sirs OR sofa OR qsofa OR "Sequential Organ Failure Assessment") OR AB=(sepsis OR septic* OR "Systemic Inflammatory Response Syndrome" OR sirs OR sofa OR qsofa OR "Sequential Organ Failure Assessment")

1. AI: 3,038,660

TS=("Artificial Intelligence" OR ai OR "Computational Intelligence" OR "Machine Intelligence" OR "Machine Learning" OR "Deep Learning" OR "Neural Network" OR "Natural Language Processing" OR "Decision Tree*" OR "Random Forest" OR "Support Vector Machine" OR "Gradient Boosting" OR "Bayes" OR "Adaboosting" OR "Regression") OR TI=("Artificial Intelligence" OR ai OR "Computational Intelligence" OR "Machine Intelligence" OR "Machine Learning" OR "Deep Learning" OR "Neural Network" OR "Natural Language Processing" OR "Decision Tree*" OR "Random Forest" OR "Support Vector Machine" OR "Gradient Boosting" OR "Bayes" OR "Adaboosting" OR "Regression") OR AB=("Artificial Intelligence" OR ai OR "Computational Intelligence" OR "Machine Intelligence" OR "Machine Learning" OR "Deep Learning" OR "Neural Network" OR "Natural Language Processing" OR "Decision Tree*" OR "Random Forest" OR "Support Vector Machine" OR "Gradient Boosting" OR "Bayes" OR "Adaboosting" OR "Regression")

1. Emergency Department: 891,913

TS=("Emergency Department*" OR "Emergency Room*" OR "Emergency Service*" OR "Emergency Ward*" OR "Emergency Unit*" OR "Emergency Hospital*" OR "Emergency Medical Service*" OR "Emergency Patient*" OR "Emergency Admiss*" OR "Emergency Admit*" OR "Accident and Emergency" OR emergenc*) OR TI=("Emergency Department*" OR "Emergency Room*" OR "Emergency Service*" OR "Emergency Ward*" OR "Emergency Unit*" OR "Emergency Hospital*" OR "Emergency Medical Service*" OR "Emergency Patient*" OR "Emergency Admiss*" OR "Emergency Admit*" OR "Accident and Emergency" OR emergenc*) OR AB=("Emergency Department*" OR "Emergency Room*" OR "Emergency Service*" OR "Emergency Ward*" OR "Emergency Unit*" OR "Emergency Hospital*" OR "Emergency Medical Service*" OR "Emergency Patient*" OR "Emergency Admiss*" OR "Emergency Admit*" OR "Accident and Emergency" OR emergenc*)

1. Prediction: 18,206,892

TS=(predict* OR diagnos* OR prognos* OR detect* OR identif*) OR TI=(predict* OR diagnos* OR prognos* OR detect* OR identif*) OR AB=(predict* OR diagnos* OR prognos* OR detect* OR identif*)

1. Combination: 989

1.2.4 Search in MEDLINE (via Ovid)

1. Sepsis: 228,928

(Sepsis or "Systemic Inflammatory Response Syndrome").sh. or (Sepsis or Septic* or "Systemic Inflammatory Response Syndrome" or SIRS or SOFA or qSOFA or "Sequential Organ Failure Assessment").ti,ab,kw.

1. AI: 1,740,756

(Algorithms or "Artificial Intelligence"or "Machine Learning"or "Natural Language Processing" or "Neural Networks, Computer").sh. or ("Artificial Intelligence" or AI or "Computational Intelligence" or "Machine Intelligence" or "Machine Learning" or "Deep Learning" or "Neural Network" or "Natural Language Processing" or "Decision Tree*" or "Random Forest" or "Support Vector Machine" or "Gradient Boosting" or "Naïve Bayes" or "Adaboosting" or "Regression").ti,ab,kw.

1. Emergency Department: 574,399

("Emergency Medical Services" or "Emergency Medicine").sh. or ("Emergency Department*" or "Emergency Room*" or "Emergency Service*" or "Emergency Ward*" or "Emergency Unit*" or "Emergency Hospital*" or "Emergency Medical Service*" or "Emergency Patient*" or "Emergency Admiss*" or "Emergency Admit*" or "Accident and Emergency" or emergenc*).ti,ab,kw.

1. Prediction: 10,802,038

(Diagnosis or Prognosis).sh. or (Predict* or Diagnos* or Prognos* or Detect* or Identif*).ti,ab,kw.

1. Combination: 838

1.2.5 Search in Embase (via Ovid)

Different from the MeSH term, we check the subject headings in the Emtree term list for retrieval.

1. Sepsis: 394,396

(Sepsis or "Systemic Inflammatory Response Syndrome").sh. or (Sepsis or Septic* or "Systemic Inflammatory Response Syndrome" or SIRS or SOFA or qSOFA or "Sequential Organ Failure Assessment").ti,ab,kw.

1. AI: 2,066,675

("Artificial Intelligence" or "Machine Learning" or "Natural Language Processing" or "Decision Tree" or "Big Data").sh. or ("Artificial Intelligence" or AI or "Computational Intelligence" or "Machine Intelligence" or "Machine Learning" or "Deep Learning" or "Neural Network" or "Natural Language Processing" or "Decision Tree*" or "Random Forest" or "Support Vector Machine" or "Gradient Boosting" or "Naïve Bayes" or "Adaboosting" or "Regression").ti,ab,kw.

1. Emergency Department: 891,572

("Emergency Health Service" or "Emergency Medicine" or "Emergency Ward" or "Hospital Emergency Service").sh. or ("Emergency Department*" or "Emergency Room*" or "Emergency Service*" or "Emergency Ward*" or "Emergency Unit*" or "Emergency Hospital*" or "Emergency Medical Service*" or "Emergency Patient*" or "Emergency Admiss*" or "Emergency Admit*" or "Accident and Emergency" or emergenc*).ti,ab,kw.

1. Prediction: 15,008,819

("Prediction" or "Predictive Model" or "Diagnosis" or "Prognosis").sh. or (Predict* or Diagnos* or Prognos* or Detect* or Identif*).ti,ab,kw.

1. Combination: 1,660

##### Supplementary 2: Screening Criteria

###### 2.1 The Screening Criteria Table (S2 & S3)

**Table S2. Data screening table.**

| **No.** | **Topic** | **Questionnaire** | **Yes → Proceed** | **NO → Exclude** |
| --- | --- | --- | --- | --- |
| 1 | Publication Status | Was the study published in a scholarly (peer-reviewed) journal? | ☐ | ☐ |
| 2 | Language | Was the study published in English? | ☐ | ☐ |
| 3 | Years Considered | Is the study published from 2019 onwards? | ☐ | ☐ |
| 4 | Study Design | The study was not a case report or review article (including systematic review, narrative review, scoping review, rapid review, umbrella review). | ☐ | ☐ |
| 5 | Study Setting | Was the study conducted in an emergency department environment? This setting can be replaced by phrases "emergency + room/ (medical) service/ ward/ unit/ hospital/ admiss*/ admit*/ patient/ physician", "casualty department" and "accident and emergency". | ☐ | ☐ |
| 6 | Intervention | Did the study utilize Artificial Intelligence tools or algorithms? These could include Machine Learning (Decision Trees (DT)/ Random Forest (RF)/ Support Vector Machine (SVM)/ Logistic Regression (LR)/ Adaboosting/ Gradient Boosting (GB)/ Naïve Bayes (NB)/ k-Nearest Neighbor (kNN)), Deep Learning (Long Short-Term Memory (LSTM) Networks/ Conventional Neural Network (CNN)/ Gated Recurrent Unit (GRU)/ Neural Network (NN)/ Multitask Gaussian Process (MGP)/ Temporal Conventional Network (TCN)/ Recurrent Neural Network (RNN)/ CNN-LSTM/ CNN-GRU/ MGP-RNN/ RNN-GRU/ SepLSTM), and Natural Language Processing (NLP). | ☐ | ☐ |
| 7 | Purpose | Did this study focus on the sepsis prediction and mention it in its title or abstract? | ☐ | ☐ |
| 8 | Population | Did the study involve human patients in above study setting? | ☐ | ☐ |
| 9 | Outcome | Did the study consider the effectiveness of AI models for sepsis prediction (including AUROC, accuracy, sensitivity and specificity)? | ☐ | ☐ |
|  | **Final Decision** |  | ☐ Include | ☐ Exclude |

Instruction for reviews:

Please review each study based on the above criteria and check "Yes" or "No" as appropriate.

Studies must meet all "Yes" criteria for inclusion in the review.

The "Final Decision" section is used to indicate whether the study will be included or excluded based on the screening criteria.

**Table S3. Eligibility criteria table.**

| **Eligibility Criteria** | |
| --- | --- |
| **Inclusion Criteria** | |
| IC1 | Studies published in peer-reviewed journals to mitigate the unreviewed bias |
| IC2 | Studies written in English to facilitate accessibility |
| IC3 | Studies published from 2019 onwards to ensure that AI technology reviewed reflects the latest developments |
| IC4 | Studies that mentioned sepsis prediction in their titles or abstracts |
| IC5 | Studies applying AI tools or algorithms, such as Machine Learning (ML) or Deep Learning (DL), and these methods should be highlighted in the titles or abstracts |
| IC6 | Studies conducted in emergency department facilities instead of non-ED or outpatient settings |
| IC7 | Studies that report on the effectiveness of AI models for sepsis prediction like performance measures, results may also be related to the predictive timing, compared outcomes with traditional prediction methods |
|  |  |
| **Exclusion Criteria** | |
| EC1 | Studies classified as case reports or review articles, such as systematic reviews, narrative reviews, scoping reviews, rapid reviews and umbrella reviews |
| EC2 | Studies did not focus on the prediction of sepsis |
| EC3 | Studies mainly focused on non-human subjects or experimental research unrelated to human healthcare |
|  |  |

###### 2.2 Classical AI algorithms

Machine Learning (ML):

Decision Trees (DT), Random Forest (RF), Support Vector Machine (SVM), Logistic Regression (LR), Adaptive Boosting (AdaBoost), Gradient Boosting (GB), Naïve Bayes (NB), k-Nearest Neighbor (KNN), etc.

Deep Learning (DL):

Long Short-Term Memory (LSTM) Networks, Conventional Neural Network (CNN), Gated Recurrent Unit (GRU), Neural Network (NN), Multitask Gaussian Process (MGP), Temporal Conventional Network (TCN), Recurrent Neural Network (RNN), CNN-LSTM, CNN-GRU, MGP-RNN, RNN-GRU, etc.

Natural Language Processing (NLP)

##### Supplementary 3: Data Extraction Results

###### 3.1 Study Characteristics

**Table S4. Study characteristics.**

| **Author** | **Year of Publication** | **Country** | **Clinical Setting** | **Demographic** | **Sepsis Definition** | **Clinical Comparator** | **Research Objective** | **Study Period** |
| --- | --- | --- | --- | --- | --- | --- | --- | --- |
| Delahanty et al. | 2019 | United States | EDs at 49 urban community hospitals operated by Tenet Healthcare | Adult patients | Rhee sepsis criteria | SIRS/SOFA/qSOFA/MEWS/NEWS | Early prediction of sepsis | 2016 - 2017 |
| Perng et al. | 2019 | Taiwan, China | ED at the Kaohsiung Chang Cheng Memorial Hospital | Adult patients | Infection | qSOFA | Mortality prediction of sepsis | 2007 - 2013 |
| Kim et al. | 2020 | South Korea | ED at a tertiary academic hospital in South Korea | Adult patients (≥20 years old) | Sepsis-3 | qSOFA/MEWS | Septic shock prediction | 2008 - 2016 |
| Mohamed et al. | 2020 | United States | ED at Detroit Medical Center | No Limitation | ICD-9-CM | Not reported | Early prediction of sepsis | 1 year |
| Zhao et al. | 2020 | China | EDs from MIMIC-III | Adult patients | Sepsis-3 | SOFA | Mortality prediction of sepsis | 2001 - 2012 |
| Kwon et al. | 2020 | South Korea | EDs at four hospitals of the Hallym University Medical Center | Adult patients | Sepsis-3 | qSOFA/MEWS | Mortality prediction of sepsis | 2016 - 2018 |
| Bedoya et al. | 2020 | United States | ED at single tertiary academic medical center | Adult patients | SIRS + Blood culture order + End-organ failure | qSOFA/NEWS | Early prediction of sepsis | 2014 - 2015 |
| Scott et al. | 2021 | United States | EDs and urgent care sites within a regional pediatric healthcare system | Children aged 60 days to 17 years old | Infection | Not reported | Septic shock prediction | 2013 - 2018 |
| van Doorn et al. | 2021 | The Netherlands | ED at the Maastricht University Medical Center | Adult patients | Infection + SIRS/SOFA | SOFA/REMS/MEDS | Mortality prediction of sepsis | 2015 - 2016 |
| Zhang et al. | 2021 | United States | EDs participating in the 2019 DII National Data Science Challenge | Adult patients | Sepsis-2 + SIRS | qSOFA/MEWS/NEWS | Early prediction of sepsis | 2000 - 2016 |
| Taneja et al. | 2021 | United States | EDs at Carle Foundation Hospital and OSF Saint Francis Medical Center in Illinois | Adult patients | Sepsis-3 | SOFA | Early prediction of sepsis | 2018 - 2019 |
| Ehwerhemuepha et al. | 2021 | United States | ED at a tertiary pediatric institution | Pediatric patients | ICD-9-CM + ICD-10-CM | Not reported | Sepsis prediction and mortality prediction | 2013 - 2019 |
| Wardi et al. | 2021 | United States | EDs at two academic medical centers (UCSD and Emory University in Atlanta) | Adult patients | Sepsis-3 + CMS | Not reported | Septic shock prediction | 2014 - 2019 |
| Yun et al. | 2021 | South Korea | ED at a tertiary care academic center | Adult patients | Sepsis-3 | NEWS | Septic shock prediction | 2014 - 2018 |
| Karlsson et al. | 2021 | Sweden | ED at Södersjukhuset in Stockholm | Adult patients | Sepsis-2 + ICD-10-CM | Not reported | Mortality prediction of sepsis | 2013 |
| Shashikumar et al. | 2021 | United States | EDs at the University of California San Diego Health and Emory University Hospital | Adult patients | Sepsis-3 | Not reported | Early prediction of sepsis | 2016 - 2020 |
| Lin et al. | 2021 | Taiwan, China | EDs at Chi-Mei Medical Center and Taoyuan General Hospital | Adult patients (≥20 years old) | Sepsis-3 | qSOFA | Early prediction of sepsis | 2016 - 2018 |
| Chao et al. | 2022 | Taiwan, China | EDs at three teaching hospitals in Northern Taiwan | Adult patients | Sepsis-3 | SOFA | Mortality prediction of sepsis | 2014 - 2017 |
| Kijpaisalratana et al. | 2022 | Thailand | ED at an urban, university-affiliated hospital | Adult patients | Sepsis-3 + ICD-10-CM | qSOFA/MEWS | Early prediction of sepsis | 2018 - 2020 |
| Choi et al. | 2022 | South Korea | ED of a tertiary teaching hospital | Adult patients with fever | Infection | Not reported | Septic shock prediction | 2020 - 2021 |
| Cheng et al. | 2022 | Taiwan, China | EDs in Taiwan | Adult patients | Sepsis-3 | qSOFA/MEWS/REMS/MEDS | Mortality prediction of sepsis | 2006 - 2017 |
| Aguirre et al. | 2022 | Spain | ED at Galdakao-Usansolo Hospital | Adult patients with fever | qSOFA ≥2 | Not reported | Early prediction of sepsis | 2021 |
| Chiu et al. | 2022 | Taiwan, China | ED at a single medical center in Taiwan | No Limitation | Infection | SOFA | Prognosis of sepsis | 2017 - 2020 |
| Mercurio et al. | 2023 | United States | ED at a tertiary care children's hospital | Pediatric patients (≤18 years) | Improving Pediatric Sepsis Outcomes (IPSO) Collaborative criteria | Not reported | Early prediction of sepsis | 2017 - 2019 |
| Greco et al. | 2023 | Italy | ED at IRCCS Humanitas Research Hospital | Adult patients | Angus/AHRQ criteria + ICD-9-CM | SOFA/qSOFA | Mortality prediction of sepsis | over 18 months |
| Guo et al. | 2023 | China | EDs at Peking Union Medical College Hospital and Beijing Tsinghua Changgung Hospital | Adult patients | Sepsis-3 | NEWS | Early prediction of sepsis | 2017 - 2021 |
| Jeon et al. | 2023 | South Korea | ED at a tertiary care hospital in Korea | Adult patients | Sepsis-3 | SOFA/qSOFA/MEWS/NEWS | Mortality prediction of sepsis | 2016 - 2020 |
| Prasad et al. | 2023 | United States | EDs at Massachusetts General Hospital and three additional hospitals | Adult patients | Rhee sepsis criteria | qSOFA | Early prediction of sepsis | 2014 - 2018 |
| Wong et al. | 2023 | Hong Kong, China | ED at the Queen Mary Hospital | Adult patients | Sepsis-3 | qSOFA | Mortality prediction of sepsis | 2021 - 2022 |
| Brann et al. | 2024 | United States | EDs at four academically affiliated hospitals | Adult patients | Health system sepsis committee criteria + US Centers for Medicare & Medicaid Services toolkit criteria | Not reported | Early prediction of sepsis | 2015 - 2021 |
| Park et al. | 2024 | South Korea | EDs across 19 hospitals | Adult patients (≥19 years) | Sepsis-3 | Not reported | Mortality prediction of sepsis | 2019 - 2020 |
| Aygun et al. | 2024 | Turkey | EDs from an open-access dataset | Adult patients | Sepsis-3 | Not reported | Early prediction of sepsis |  |
| Xia et al. | 2024 | China | EDs at West China Hospital, Sichuan University, Chengdu First People's Hospital and Chengdu Shangjin Nanfu Hospital | Acute pancreatitis patients (≥14 years old) | Sepsis-3 | qSOFA | Early prediction of sepsis | 2017 - 2019 |
| Hou et al. | 2024 | Taiwan, China | ED at Taipei Tzu Chi Hospital | Adult patients | Sepsis-3 | SOFA/qSOFA/NEWS | Early prediction of sepsis | 2020 - 2022 |
| Xie et al. | 2024 | China | EDs from MIMIC-IV | No Limitation | Sepsis-3 | Not reported | Early prediction of sepsis | 2008 - 2019 |
| Song et al. | 2024 | China | EDs from MIMIC-IV-ED | Adult patients | Infection + SOFA | MEWS/NEWS/REMS | Early prediction of sepsis | 2011 - 2019 |

###### 3.2 Data Preparation

**Table S5. Data preparation (part A).**

| **Author** | **Data Source** | **Collection Method** | **Detailed Collection** | **Sample Size** | **Training Dataset** | **Validation Dataset** | **Testing Dataset** | **Positive Patients** | **Sepsis Prevalence** |
| --- | --- | --- | --- | --- | --- | --- | --- | --- | --- |
| Delahanty et al. | EHR | Retrospective cohort study | Automatic EHR extraction | 2,759,529 | 1,839,503 (66.7%) |  | 920,026 (33.3%) | 54,661 | 1.98% |
| Perng et al. | Chang Gung Research Database | Retrospective cohort study | Automatic data extraction | 42,220 | 70% |  | 30% |  | 72 h: 4.7% 28 days: 14.1% |
| Kim et al. | EHR | Retrospective single-center observational study | Automatic EHR extraction | 49,560 | 60% |  | 40% | 4,817 | 9.72% |
| Mohamed et al. | EHR | Retrospective cohort study | Automatic EHR extraction + adjudication by sepsis expert physician | 1,887 |  |  |  | 912 | 48.33% |
| Zhao et al. | MIMIC-III database | Retrospective cohort study | Automatic MIMIC-III extraction | 5,663 | 3964 (70%) | 1,699 (30%) |  | 1,182 | 20.87% |
| Kwon et al. | EHR | Retrospective multi-center study | Automatic EHR extraction + Smart CDW for collecting clinical data + ED nursing assessment | 23,587 | 74% | 8% | 4,234 (18%) | 941 | 3.99% |
| Bedoya et al. | EHR | Retrospective single-center cohort study | Automatic EHR extraction | 42,979 | 80% | 10% | 4,297 (10%) | 8,160 | 18.99% |
| Scott et al. | EHR | Retrospective observational study | Automatic EHR extraction + Vital and lab measures collection after arrival | 2,318 | 1,594 |  | 724 | 197 | 8.50% |
| van Doorn et al. | EHR + Laboratory information system | Retrospective single-center cohort | Automatic data extraction | 1,344 |  |  | 100 | 174 | 12.95% |
| Zhang et al. | EHR | Retrospective cohort study | Automatic EHR extraction | 178,843 | 80% | 10% | 10% | 52,802 | 29.52% |
| Taneja et al. | EHR | Prospective observational cohort study | Clinical sample collection + Biomarker measurement + Automatic EHR extraction | 1,400 | 933 (2/3) |  | 467 (1/3) | 410 | 29.29% |
| Ehwerhemuepha et al. | EHR | Retrospective cohort | Automatic EHR extraction | 537,837 | 50% | 15% | 35% | 1,610 | 0.30% |
| Wardi et al. | EHR | Retrospective multi-center cohort study | Automatic EHR extraction | 183,573 | 80% | 20% | 48,252 | 14,908 | 8.12% |
| Yun et al. | EHR | Retrospective cohort study | Automatic EHR extraction | 41,687 | 33,349 (80%) | 8,338 (20%) |  | 672 | 1.61% |
| Karlsson et al. | EHR | Retrospective cross-sectional study | Automatic EHR extraction | 445 | 356 (80%) | 89 (20%) |  | 98 | 22.02% |
| Shashikumar et al. | EHR | Retrospective + prospective cohort | Automatic EHR extraction + Real-time data was organized into time series bins | 109,053 | 80% | 20% | 18,148 | 9,927 | 9.10% |
| Lin et al. | EHR | Retrospective cohort study | Automatic EHR extraction | 10,040 | 6,637 (80%) | 1,659 (20%) | 1,744 | 2,258 | 22.49% |
| Chao et al. | EHR | Prospective multi-center cohort study | Experimental data acquisition + Scoring system calculations + PCPs and telephone surveys + Automatic EHR extraction | 555 | 389 (70%) |  | 166 (30%) | 101 | 18.20% |
| Kijpaisalratana et al. | EHR | Retrospective single-center cohort | Automatic EHR extraction + chief complaint obtained from triage nursing notes | 133,707 | 60% | 20% | 20% | 1,203 | 0.90% |
| Choi et al. | Data from wearable wireless devices | Retrospective study | Continuous monitoring and manual measurements + Cloud-based upload and stored in EHR + quality-checked in real-time by researchers | 468 | 277 (60%) | 93 (20%) | 98 (20%) | 76 | 16.24% |
| Cheng et al. | EHR | Retrospective observational cohort study | Automatic EHR extraction | 193,646 | 60% | 20% | 20% | 19,434 | 10.04% |
| Aguirre et al. | Laboratory Data | Prospective observational study | CBC from Mindray BC-6800 Plus analyzer + Recorded CPD + retrieved WBC & NLR | 698 | 70% | 30% |  | 272 | 38.97% |
| Chiu et al. | EHR | Retrospective + prospective cohort | Automatic EHR extraction + Lab results collection | 9,397 |  |  | 2,603 | 568 | 6.04% |
| Mercurio et al. | EHR | Retrospective observational study | Automatic EHR extraction | 35,074 | 80% |  | 20% | 191 | 0.54% |
| Greco et al. | EHR | Retrospective cohort study | Automatic EHR extraction | 425 | 90% | 10% |  | 65 | 15.29% |
| Guo et al. | EHR | Retrospective cohort study | Automatic EHR extraction | 1,840 | 1272 (70%) | 568 (30%) |  | 478 | 25.98% |
| Jeon et al. | Registry of sepsis patients | Retrospective cohort study | Automatic sepsis patient registry extraction | 810 | 607 (75%) |  | 203 (25%) | 259 | 31.98% |
| Prasad et al. | EHR | Retrospective cohort study | Automatic EHR extraction + chart reviews collection | 1,663 | 1,164 (70%) | 499 (30%) | 784 | 894 | 53.76% |
| Wong et al. | EHR | Retrospective observational study | Automatic EHR extraction from clinical management system of the hospital authority | 558 | 70% |  | 30% | 144 | 25.81% |
| Brann et al. | EHR | Retrospective cohort study | vital sign measurements + and acuity scores + free-text nursing triage notes (into EHR) | 1,059,386 | 950,921 (90%) |  | 108,465 (10%) | 35,318 | 3.33% |
| Park et al. | Nationwide Korean Sepsis Alliance cohort data (from EHR) | Prospective multi-center cohort study | Automatic EHR extraction | 5,112 | 4,089 (80%) |  | 1,023 (20%) | 1,455 | 28.46% |
| Aygun et al. | An open-access dataset | Prospective observational study | Automatic data extraction | 1,572 | 80% |  | 20% | 560 | 35.62% |
| Xia et al. | EHR + bedside parameters | Retrospective multi-center cohort | Bedside data analysis + Automatic EHR extraction | 771 | 539 (70%) |  | 232 (30%) | 559 | 72.50% |
| Hou et al. | EHR | Retrospective cohort study | Automatic EHR extraction + S-CDSS captured real-time data | 70,758 | 58,965 | 27,545 | 11,793 |  |  |
| Xie et al. | MIMIC-IV database | Retrospective cohort study | Automatic data extraction | 16,619 | 90% |  | 10% | 8,219 | 49.46% |
| Song et al. | MIMIC-IV-ED database | Retrospective single center cohort study | Automatic data extraction | 425,737 | 80% |  | 20% | 13,106 | 3.08% |

**Table S5. Data preparation (part B).**

| **Author** | **Data Preprocessing Method (Cleaning)** | **Data Preprocessing Method** | **Feature Engineering** | **Total Feature Number** | **Final Feature Number** | **Feature Type** | **Feature Importance** |
| --- | --- | --- | --- | --- | --- | --- | --- |
| Delahanty et al. | ① Missing value processing: extreme value imputation | ① Data splitting: training : testing = 66.7% : 33.3% | ① Feature generation: New feature inclusion | 217 | 13 | vital, lab, maximum lactic acid, shock index multiplied by age | Lactic acid, Shock index × age, WBC, Neutrophils, Glucose, BUN, RR, Albumin, SBP, Creatinine, BT |
| Perng et al. | ① Missing value processing: Median imputation | ① Data transformation: Standardization (Z-score) ② Data splitting: training : testing = 70% : 30% | ① Feature extraction: AE + PCA | 53 | 53 | demographic, vital, lab | RDW, GCS |
| Kim et al. | ① Missing value processing: Mean/mode imputation | ① Data transformation: Yeo-Johnson method ② Data encoding: One-hot encoding ③ Data balancing: Ensemble method ④ Data splitting: training : testing = 60% : 40% | ① Feature selection: Embedded ② Feature extraction: SVD, t-SNE | 42 | 27 | demographic, vital, lab, chief complaint, consciousness | SBP, Alert, SO2, DBP, BT, Neutrophils, Monocytes, WBC, Lymphocytes, Creatinine |
| Mohamed et al. | ① Missing value processing: Using nominal values | ① Data balancing: Ensemble method | ① Feature extraction | 43 | 43 | demographic, vital, lab, nursing assessments |  |
| Zhao et al. | ① Outlier processing: Delection | ① Data splitting: training : validation = 70% : 30% | ① Feature selection: Filter + Wrapper + Embedded (GBM) |  |  | demographic, vital, lab, comorbidity, intervention | RDW, NLR, Platelet-to-lymphocyte ratio, Lymphocyte-to-monocyte ratio, Age |
| Kwon et al. |  | ① Data transformation: Normalization (Min-Max scaling) ② Data balancing: Oversampling (SMOTE) ③ Data splitting: training : validation : testing = 74% : 8% : 18% | ① Feature selection: Embedded (GBM/RF) | 15 | 15 | demographic, vital, lab, clinical data | Age, Gender, ED diagnosis, SBP, RR, Mental status, BT, HR, Partial pressure of carbon dioxide, WBC, Hospitalization, ICU, Mechanical ventilation, Mortality |
| Bedoya et al. | ① Missing value processing: MGP imputation | ① Data splitting: training : validation : testing = 80% : 10% : 10% | ① Feature extraction | 86 |  | demographic, vital, lab, comorbidity, medication |  |
| Scott et al. | ① Missing value processing: Delection |  | ① Feature selection: Embedded | 76 | 20 | demographic, vital, lab, medical history | SBP, DBP, Age, HR, SI, RR, Oncological comorbidity, Hospitalization, Central venous catheter, LAC, Albumin, Alanine transaminase, Bilirubin, BUN, Creatinine, Glucose, Neutrophils, WBC, Hemoglobin |
| van Doorn et al. | ① Missing value processing: Additional 'absence/presence' replacement |  | ① Feature selection: Embedded (XGB) | 106 |  | demographic, vital, lab | HR, Blood group, BUN, Albumin, Magnesium, PLT, GCS, SO2, Age, BT, Glucose, SBP, Creatinine, Lab amount, CRP |
| Zhang et al. | ① Missing value processing: Using embedding or imputing available admission data | ① Data encoding: Event embedding, time encoding ② Data splitting: training : validation : testing = 80% : 10% : 10% | ① Feature selection: Embedded ② Feature extraction | 113 |  | demographic, vital, lab, medical history, clinical data | RR, PR, GCS, DBP, SBP, Fraction of inspired oxygen, HR, MAP, SO2 |
| Taneja et al. | ① Missing value processing: Median imputation | ① Data splitting: training : testing = 2 : 1 | ① Feature selection: Embedded | 24 | 24 | demographic,vital, lab | Procalcitonin, IL-6, BUN, Albumin, Creatinine, Bilirubin, Neutrophils, GCS, SBP, PLT, CRP, WBC, Monocytes, DBP, Potassium, LAC, BT, RR, Sodium, Glucose, HR, SO2, Age |
| Ehwerhemuepha et al. | ① Missing value processing: Automatically handled by GBM | ① Data splitting: training : validation : testing = 50% : 15% : 35% | ① Feature selection: Embedded ② Feature generation: New feature inclusion | 46 | 46 | demographic, vital, lab, medical history | Age, HR, Hospitalization, BT, SBP, Previous sepsis, Medications, Antibiotics, DBP, RR, Carbapenem, SO2 |
| Wardi et al. | ① Missing value processing: Mean imputation | ① Data transformation: Standardization ② Data splitting: training : validation = 80% : 20% | ① Feature selection: Embedded | 40 | 20 | demographic, vital, lab, comorbidity, medication | SBP, RR, HR, BUN, DBP, Fraction of inspired oxygen, Magnesium, Potassium, BT, MAP, pH, Aspartate aminotransferase |
| Yun et al. | ① Missing value processing: Deletion | ① Data splitting: training : validation = 80% : 20% | ① Feature selection: Embedded | 9 | 9 | demographic, vital, lab, disposition | Gender, Age, SBP, DBP, PR, RR, BT, SO2, NEWS score |
| Karlsson et al. | ① Missing value processing: Deletion | ① Data balancing: Undersampling, ensemble method ② Data splitting: training : validation = 80% : 20% | ① Feature selection: Embedded | 91 | 91 | demographic, vital, lab, symptom, arrival mode | Fever, Chills, SO2, EMS arrival, Abnormal behavior or councioussness, Abnormal verbal response, Breathing difficulties |
| Shashikumar et al. | ① Missing value processing: Sample-and-Hold, mean imputation | ① Data transformation: Standardization (Z-score) ② Data balancing: Resampling | ① Feature extraction | 40 | 40 | demographic, vital, lab | Elapsed time, BT, HR, WBC, Alkalinephos, LAC, BUN, PLT, Age, SBP, RR |
| Lin et al. | ① Missing value processing: Automatically handled by XGBoost | ① Data splitting: training : validation = 80% : 20% | ① Feature selection: Embedded | 32 | 19 | demographic, vital, lab | Complete blood picture, Sodium, Lymphocytes, Creatinine, BT, PLT, RDW, Glutamate pyruvate transaminase, Hemoglobin, Neutrophils, WBC, Glucose, LAC, HR |
| Chao et al. | ① Missing value processing: Median imputation for continuous variables, mode imputation for categorical variables | ① Data transformation: Standardization ② Data splitting: training : testing = 70% : 30% | ① Feature selection: Wrapper + Embedded | 219 | 30 | demographic, vital, lab, disease severity | SOFA score, IL-8, D-dimer, IL-6, Angiopoietin-2, Albumin, PR, Fibrin degradation product, Cortisol, SO2, PLT, E-selection, Bicarbonate, LAC, Uric acid, Vascular cell adhesion protein 1, Procalcitonin |
| Kijpaisalratana et al. | ① Missing value and outlier processing: Mean/median imputation | ① Data encoding: One-hot Encoding ② Data balancing: Oversampling (SMOTE) ③ Data splitting: training : validation : testing = 60% : 20% : 20% | ① Feature selection: Embedded ② Feature extraction: TF-IDF | 15 | 15 | demographic, vital, comorbidity, arrival mode, emergency severity index, chief complaint | BT, Age, Fever, SI, PR, GCS, RR, SBP, Mental status, DBP, SO2, Emergency severity index |
| Choi et al. | ① Missing value processing: Median/carry forward imputation ② Outlier processing: Deletion | ① Data splitting: training : validation : testing = 60% : 20% : 20% | ① Feature extraction | 14 | 14 | demographic, vital | DBP, SBP, Age, HR, BT, SO2, RR, Gender |
| Cheng et al. | ① Missing value processing: forward-filled ② Outlier processing: deletion | ① Data transformation: Normalization (Min-Max scaling), standardization (Z-score) ② Data splitting: training : validation : testing = 60% : 20% : 20% | ① Feature extraction | 7 | 7 | demographic, vital | Age, Gender, SBP, DBP, HR, RR, BT |
| Aguirre et al. |  | ① Data transformation: Standardization ② Data splitting: training : validation = 70% : 30% | ① Feature extraction | 12 | 12 | lab | Lymphocytes, NLR, Neutrophils, Monocytes, WBC |
| Chiu et al. | ① Missing value processing: MissForest | ① Data transformation: Standardization ② Data balancing: Class Weight Adjustment | ① Feature selection: Filter ② Feature extraction | 30 | 24 | demographic, vital, lab, medical history, ED management | RR, Hemoglobin, Malignancy, SI, GCS, Low flow O2, Age, PR, Potassium, DBP, SBP, CRP, Creatinine, Neutrophils, WBC, Fluid challenge |
| Mercurio et al. | ① Missing value processing: Standard age-specific range imputation (vital signs); '0' encoding (clinical scores) | ① Data transformation: Normalization ② Data balancing: Oversampling (SMOTE) ③ Data splitting: training : testing = 80% : 20% | ① Feature selection: Filter | 76 |  | demographic, vital, lab, clinical scoring, intake/output, sociodemographic, patient problem list, medication | HR, MAP, SO2, SBP, DBP, BT, Age, RR, Immunization status |
| Greco et al. | ① Missing value processing: missRanger | ① Data balancing: Data balancing: Oversampling (SMOTE) ② Data splitting: training : validation = 90% : 10% | ① Feature selection: Embedded | 44 |  | demographic, vital, lab, comorbidity, clinical data | Sodium, HR, Glucose, Age, SBP, Bicarbonate, BT, MAP, Bilirubine, pO2 |
| Guo et al. | ① Outlier/missing value processing: deletion | ① Data transformation: Discretization ② Data splitting: training : validation = 70% : 30% |  | 48 | 48 | demographic, vital, lab, comorbidity | Alcohol consumption, Lung infection, BT, RR, HR, BUN, WBC |
| Jeon et al. | ① Missing value processing: MICE | ① Data transformation: Normalization (Min-Max scaling) ② Data encoding: One-hot encoding | ① Feature selection: Wrapper (RFE) + Embedded (GBM) | 55 | 33 | demographic, vital, lab | Septic shock, LAC, Malignancy, Age, SO2, pH, BT, RR |
| Prasad et al. | ① Missing value processing: Sample-and-Hold, mean imputation | ① Data transformation: Normalization ② Data splitting: training : validation = 70% : 30% | ① Feature selection: Embedded (L2, variance selection) ② Feature generation: New feature inclusion | 24 | 24 | demographic, vital, medical history, symptoms |  |
| Wong et al. | ① Missing values processing: Admission data imputation or deletion | ① Data transformation: Normalization ② Data splitting: training : testing = 70% : 30% | ① Feature selection: Filter + Embedded ② Feature extraction ③ Feature generation: New feature inclusion | 12 | 12 | demographic, vital, lab, medical history | RR, SO2, Hemoglobin, PR, Age, Charlson comorbidity index, MAP, Gender, NLR, Old age home resident status, Platelet-to-lymphocyte ratio, RDW |
| Brann et al. | ① Missing value processing: deletion | ① Data encoding: Vectorization ② Data balancing: Class Weight Adjustment, ensemble method ③ Data splitting: training : testing = 70% : 30% | ① Feature selection: Embedded ② Feature extraction: PCA | 12 | 12 | demographic, vital, lab, triage note | WBC, BT, Creatinine, SO2, HR, Lactic acid, PLT, Triage note |
| Park et al. | ① Outlier/missing value processing: deletion | ① Data transformation: Normalization (Min-Max scaling) ② Data splitting: training : testing = 70% : 30% | ① Feature selection: Filter + Embedded | 213 | 44 | demographic, vital, lab, comorbidity, infection | Albumin, LAC, Infection Site, Prothrombin time-international normalized ratio, Bilirubin, BT, PLT, Clinical frailty scale, PO2, RR, Steroid treatment, pH, Age, Hemoglobin, Charlson comorbidity index, Bilirubin, HR, Kalium, Vasopressors, CRP |
| Aygun et al. | ① Missing value processing: Imputation using random forest | ① Data balancing: Oversampling (SMOTE) ② Data splitting: training : testing = 80% : 20% | ① Feature selection: Embedded (L1) | 15 | 15 | demographic,vital, lab | Age, RR, SO2, Procalcitonin, Positive blood culture, NLR, SBP, CRP, LAC, WBC, BT, Hemoglobin, HR, SIRS, ICU |
| Xia et al. |  | ① Data balancing: Upsampling ② Data transformation: Normalization (Min-Max scaling), Standardization (Z-score) ③ Data splitting: training : testing = 70% : 30%, bootstrap | ① Feature selection: Filter (ANOVA) + Wrapper (RFE) + Embedded (RF) ② Feature extraction: PCA + PCC | 8 | 8 | vital, lab, medical history | RR, Glucose, OSI, PO2, BT, HR, LAC, Disease duration |
| Hou et al. | ① Missing value processing: Handled by LightGBM |  | ① Feature selection: Embedded | >100 | >30 | demographic,vital, lab, clinical outcomes | BT, SBP, RR, HR, Age, DBP, SO2, GCS, WBC, Neutrophils, Lymphocytes, Eosinophil, CRP, High-sensitivity cardiac troponin, Urine bacteria count, Urine protein, eGFR, Prothrombin time, Prothrombin time-international normalized ratio, Urine specific gravity, Hematocrit, Hemoglobin, BUN |
| Xie et al. | ① Missing value processing: Deletion (more than 3 missing values for 6 vitals) | ① Data encoding: Label Encoding ② Data splitting: training : testing = 90% : 10% | ① Feature generation: Synthetic feature inclusion | 13 | 13 | Demographic, vital, calculated synthetic feature | Acuity, Arrival mode, Age, SI, RR, SpO2, MAP, HR, BT, DBP, Gender, SBP, ED_Hour |
| Song et al. | ① Missing value and outlier processing: Median/mode imputation | ① Data splitting: training : testing = 80% : 20% |  | 21 | 21 | demographic, vital, triage information | ESI, Arrival mode, Age, HR, Pain scale |

###### 3.3 Model Characteristic

**Table S6. Model characteristics (part A).**

| **Author** | **Algorithm** | **Algorithm Optimization** | **AUROC (95% CI)** | **Other Output Metrics** |
| --- | --- | --- | --- | --- |
| Delahanty et al. | XGBoost | ① Training technique: 5-fold cross-validation | RoS: 1 h: 0.93 3 h: 0.95 6 h: 0.96 12 h: 0.97 24 h: 0.97  qSOFA: 0.62–0.80 SOFA: 0.78–0.90 SIRS: 0.75–0.79 MEWS: 0.62–0.79 NEWS: 0.69–0.84 | Sensitivity, Specificity, Precision |
| Perng et al. | CNN, RF, KNN, SVM | ① Training technique: K-fold cross-validation ② Optimizer: Adam optimizer | 72 h: CNN + SoftMax: 0.94 [0.94–0.94] RF: 0.89 [0.88–0.89] KNN: 0.83 [0.83–0.84] SVM: 0.93 [0.92–0.93] SIRS: 0.67 [0.67–0.68] qSOFA: 0.74 [0.73–0.74]  28-Days: CNN + SoftMax: 0.92 [0.92–0.92] RF: 0.89 [0.89–0.89] KNN: 0.84 [0.83–0.84] SVM: 0.90 [0.89–0.90] SIRS: 0.59 [0.59–0.60] qSOFA: 0.68 [0.67–0.69] | Accuracy |
| Kim et al. | MARS, SVM, GBM, RF, LASSO, RR, MLP | ① Training technique: 5-fold cross-validation ② Hyperparameter tuning: Grid search | MARS: 0.924 [0.9184–0.929] SVM: 0.914 [0.9084–0.920] GBM: 0.923 [0.9174–0.928] RF: 0.920 [0.9144–0.925] Lasso: 0.905 [0.8984–0.912] Ridge: 0.904 [0.8974–0.911] MLP: 0.911 [0.9044–0.917] Ensemble-Ave 0.902 [0.895–0.909] Ensemble-MARS: 0.902 [0.895–0.908]  qSOFA: 0.813 [0.803–0.824] MEWS: 0.790 [0.779–0.800] | Sensitivity, Specificity, AUPRC, PPV, NPV |
| Mohamed et al. | NN, SVM, DT, KNN, LR | ① Training technique: Scaled conjugate gradient backpropagation, 10-fold cross-validation ② Hyperparameter tuning: Genetic algorithm | NN: 0.97 SVM: 0.96 DT: 0.96 KNN: 0.96 LR: 0.96 | Accuracy, Sensitivity, Specificity, PPV |
| Zhao et al. | GBM, LR |  | GBM: 0.867 LR: 0.813 [0.790–0.837]  SOFA: 0.680 [0.648–0.712] OASIS: 0.685 [0.655–0.716] SAPS II: 0.744 [0.715–0.772] |  |
| Kwon et al. | XGBoost, LightGBM, RF | ① Training technique: 5-fold cross-validation ② Hyperparameter tuning: Grid search | ML: 0.86 [0.85–0.87]  qSOFA: 0.78 [0.77–0.79] SIRS: 0.68 [0.57–0.79] MEWS: 0.77 [0.67–0.86] |  |
| Bedoya et al. | MGP-RNN, RF, CR, PLR | ① Hyperparameter tuning: Hyperparameter selection using controls up to a randomly chosen time point to prevent model overfitting | MGP-RNN: 0.882 [0.870–0.894] RF: 0.836 [0.820–0.852] COX: 0.849 [0.834–0.863] PLR: 0.822 [0.805–0.836]  SIRS: 0.756 [0.739–0.772] NEWS: 0.619 [0.598–0.639] qSOFA: 0.481 [0.460–0.503] | Sensitivity, PPV |
| Scott et al. | LR |  | Temporal Test: 0.83 [0.78–0.89] Geographic Test: 0.83 [0.60–1.00] | Sensitivity, Specificity, predicted probability of hypotensive shock |
| van Doorn et al. | XGBoost, LR, RF, MLP | ① Training technique: 5-fold cross-validation, regularization mechanisms | XGB: 0.85 [0.78–0.92] LR: 0.63 [0.61–0.66] MLP: 0.66 [0.63–0.69] RF: 0.72 [0.69–0.76]  abbMEDS: 0.63 [0.54–0.73] mREMS: 0.63 [0.54–0.72] SOFA: 0.75 [0.68–0.84] Physician: 0.74 [0.65–0.82] | Accuracy, Sensitivity, Specificity, PPV, NPV |
| Zhang et al. | LSTM |  | LSTM: 0.89  MEWS: 0.63 NEWS: 0.62 SIRS: 0.62 qSOFA: 0.59 |  |
| Taneja et al. | RF | ① Training technique: 10 repeats of 5-fold cross-validation | RF: 0.83  SOFA: 0.71 SIRS: 0.83 | AUPRC, F1, Threshold, Sensitivity, Specificity, PPV, NPV |
| Ehwerhemuepha et al. | XGBoost | ① Training technique: 10-fold cross-validation ② Hyperparameter tuning: Grid search | Mortality: 0.979 [0.967–0.991] Severe sepsis: 0.990 [0.985–0.995] Non-severe sepsis: 0.976 [0.972–0.981] | Sensitivity, Specificity, PPV, NPV, relative risk, NNE, AUCPR, Probability threshold |
| Wardi et al. | CoxPh | ① Training technique: 10-fold bootstrap cross-validation | 12h: 0.833 | Sensitivity, Specificity |
| Yun et al. | XGBoost, LR, ANN | ① Training technique: 5-fold cross-validation, Adam optimizer, Dropout regularization | XGB: 0.845 [0.815–0.875] LR: 0.844 [0.815–0.874] ANN: 0.835 [0.807–0.863]  NEWS: 0.804 [0.770–0.838] | Sensitivity, Specificity, PPV, NPV, Cutoff Probability |
| Karlsson et al. | RF | ① Training technique: 10-fold cross-validation, bootstrapping | 7-day: 0.83 [0.80–0.86] 30-day: 0.80 [0.78–0.82] | Sensitivity, Specificity, PPV, NPV |
| Shashikumar et al. | COMPOSER (LR + FNN) | ① Training technique: Adam optimizer with early stopping, L1-L2 regularization ② Hyperparameter tuning: Bayesian optimization | 0.945 | Sensitivity, Specificity, PPV, NPV, DOR, FAPH |
| Lin et al. | XGBoost | ① Training technique: 5-fold cross-validation ② Hyperparameter tuning: Grid search | XGB: 0.86  SIRS: 0.68 qSOFA: 0.56 | Accuracy, Sensitivity, Specificity, PPV, NPV |
| Chao et al. | RF, XGBoost, SVM, DNN, ANN, LR | ① Training technique: 3-fold cross-validation ② Hyperparameter tuning: Grid search | RF: 0.959 [0.927–0.983] XGB: 0.934 [0.887–0.980] SVM: 0.881 [0.796–0.966] DNN: 0.846 [0.774–0.917] ANN: 0.821 [0.715–0.926] LR: 0.785 [0.642–0.929]  SOFA: [0.66–0.84] |  |
| Kijpaisalratana et al. | RF, LR, GBM, NN | ① Training technique: 5-fold cross-validation, L1-L2 regularization ② Hyperparameter tuning: Grid search (LR), random search (others) | RF: 0.931 [0.921–0.944] LR: 0.930 [0.918–0.941] GB: 0.919 [0.903–0.932] NN: 0.926 [0.914–0.937]  qSOFA: 0.635 [0.613–0.660] MEWS: 0.688 [0.662–0.715] SIRS: 0.814 [0.794–0.833] | Sensitivity, Specificity, PPV, NPV |
| Choi et al. | CNN-LSTM |  | Fragmented model: 0.858 [0.809–0.908] Accumulated model: 0.861 [0.811–0.910] | AUPRC, Sensitivity, Specificity |
| Cheng et al. | CNN, LSTM, RF | ① Training technique: 5-fold cross-validation, bootstrapping | CNN: 6 h: 0.840 12 h: 0.833 24 h: 0.807 48 h: 0.811 LSTM: 6 h: 0.761 12 h: 0.752 24 h: 0.750 48 h: 0.734 RF: 6 h: 0.770 12 h: 0.772 24 h: 0.771 48 h: 0.764  MEDS: 0.730–0.871 MEWS: 0.596–0.730 REMS: 0.620–0.800 qSOFA: 0.580–0.760 | Accuracy, Precision, Sensitivity |
| Aguirre et al. | MLP, LR, NB, KNN, SVM, RF, XGBoost | ① Training technique: 5-fold cross-validation | MLP: 0.95 [0.92–0.97] LR: 0.91 [0.88–0.95] NB: 0.91 [0.87–0.95] KNN: 0.92 [0.88–0.96] SVM: 0.94 [0.91–0.97] RF: 0.93 [0.90–0.97] XGB: 0.93 [0.90–0.96] | Accuracy, Sensitivity, Specificity, Precision, F1 |
| Chiu et al. | FNN |  | FNN: 0.85  SOFA: 0.65 | Accuracy, Sensitivity, Specificity, PPV, NPV |
| Mercurio et al. | LR, RF, CART, SVM, GNB | ① Training technique: Cross-validation | LR: 0.82 RF: 0.81 CART: 0.77 SVM: 0.81 GNB: 0.65 | Sensitivity, Specificity, Precision, F1-score, AUPRC |
| Greco et al. | RF, LR | ① Training technique: 10-fold cross-validation, holdout cross validation | RF: 0.863 LR: 0.813  qSOFA: 0.706 SOFA: 0.712 APACHE II: 0.647 | Accuracy, Sensitivity, F1, Precision |
| Guo et al. | LR |  | LR: 0.865 [0.842–0.888]  NEWS: 0.782 [0.753–0.812] |  |
| Jeon et al. | LightGBM, XGBoost, SVM, MLP, LR | ① Training technique: Leave-one-out cross-validation ② Hyperparameter tuning: Bayesian optimization | LightGBM:  7-day: 0.89 [0.84–0.94] 14-day: 0.89 [0.84–0.94] 30-day: 0.87 [0.82–0.92] XGBoost: 7-day: 0.84 [0.78–0.91] 14-day: 0.84 [0.79–0.90] 30-day: 0.84 [0.78–0.89] SVM: 7-day: 0.84 [0.78–0.90] 14-day: 0.85 [0.79–0.91] 30-day: 0.85 [0.79–0.91]  MLP: 7-day: 0.89 [0.83–0.94] 14-day: 0.88 [0.83–0.93] 30-day: 0.86 [0.81–0.92] LR: 7-day: 0.82 [0.74–0.89] 14-day: 0.84 [0.77–0.90] 30-day: 0.81 [0.74–0.88]  SOFA: 7-day: 0.68 [0.59–0.77] 14-day: 0.65 [0.57–0.73] 30-day: 0.66 [0.58–0.74] qSOFA: 7-day: 0.59 [0.52–0.66] 14-day: 0.57 [0.50–0.64] 30-day: 0.57 [0.50–0.64] NEWS: 7-day: 0.63 [0.54–0.72] 14-day: 0.63 [0.55–0.71] 30-day: 0.63 [0.55–0.71] NEWS2: 7-day: 0.62 [0.53–0.71] 14-day: 0.62 [0.54–0.71] 30-day: 0.62 [0.54–0.70] MEWS: 7-day: 0.59 [0.50–0.68] 14-day: 0.59 [0.51–0.68] 30-day: 0.57 [0.49–0.65] | AUPRC, Balanced accuracy, Brier score |
| Prasad et al. | LR | ① Training technique: L2 regularization | Bland Model: 0.77 [0.73–0.81] Essential Model: 0.83 [0.79–0.86] Full Model: 0.82 [0.78–0.86]  qSOFA: 0.63 [0.59–0.68] | Accuracy, Sensitivity, Specificity, PPV, NPV, F1 |
| Wong et al. | ANN | ① Loss function optimization: Backpropagation with gradient descent to minimize the loss function | ANN: 0.811  qSOFA: 0.694 [0.669–0.720] | Accuracy |
| Brann et al. | XGBoost | ① Training technique: L1-L2 regularization ② Hyperparameter tuning: Grid search, Bayesian optimization | Time-of-Triage Model: 0.94 Comprehensive Model: 1h: 0.94 5h: 0.96 12h: 0.97 | Sensitivity, Specificity, Macro F1, FPR |
| Park et al. | CatBoost, LR, SVM, RF, XGBoost, LightGBM | ① Training technique: 5-fold cross-validation | CAT: 0.800 [0.756–0.840] LR: 0.772 [0.726–0.815] SVM: 0.771 [0.724–0.815] RF: 0.736 [0.688–0.783] XGB: 0.797 [0.754–0.838] LGB: 0.795 [0.750–0.836] | Accuracy, Sensitivity, Specificity, Precision, F1, AUPRC |
| Aygun et al. | XGBoost, LightGBM, AdaBoost | ① Training technique: 10-fold cross-validation ② Hyperparameter tuning: Grid search | XGB: 0.940 [0.898–0.980] AdaBoost: 0.917 [0.869–0.966] LightGBM: 0.931 [0.887–0.974] | Accuracy, F1, Sensitivity, Specificity, PPV, NPV, Brier score |
| Xia et al. | RF, SVM, LDA, AE, LR-LASSO, Linear Regression, AdaBoost, DT, GP, NB | ① Training technique: 10-fold cross-validation, L2 Regularization, 'One-standard error' rule | RF: 0.947 [0.910–0.972]  qSOFA: 0.672 [0.608–0.732] APACHE II: 0.708 [0.645–0.768] BISAP: 0.680 [0.618–0.739] | Accuracy, NPV, PPV, Sensitivity, Specificity |
| Hou et al. | LightGBM | ① Training technique: 6-fold stratified cross-validation | LightGBM: 0.879 [0.859–0.900]  SIRS: 0.832 qSOFA: 0.736 SOFA: 0.830 NEWS: 0.842 | Sensitivity, Specificity, PPV, NPV |
| Xie et al. | XGBoost | ① Training technique: 10-fold cross-validation ② Hyperparameter tuning: Grid search | 0.92 [0.90–9.94] | Accuracy, Sensitivity, Specificity |
| Song et al. | XGBoost, LR, RF, AutoScore | ① Training technique: 5-fold cross-validation ② Hyperparameter tuning: Grid search | XGBoost: 0.90 [0.90–0.91] LR: 0.89 [0.89–0.90] RF: 0.90 [0.89–0.90] AutoScore: 0.89 [0.88–0.89]  NEWS: 0.67 [0.66–0.69] NEWS2: 0.66 [0.65–0.67] MEWS: 0.64 [0.63–0.65] CART: 0.74 [0.73–0.75] REMS: 0.71 [0.71–0.72] ESI: 0.79 [0.78–0.80] | Accuracy, Sensitivity, Specificity, PPV, NPV |

**Table S6. Model characteristics (part B).**

| Author | Accuracy | Sensitivity (95% CI ) | Specificity (95% CI ) | Prediction Window | Effectiveness |
| --- | --- | --- | --- | --- | --- |
| Delahanty et al. |  | 1 h: 0.677 3 h: 0.721 6 h: 0.749 12 h: 0.793 24 h: 0.846  qSOFA: 0.037–0.235 SOFA: 0.492–0.804 SIRS: 0.404–0.464 MEWS: 0.091–0.169 NEWS: 0.182–0.390 | 1 h: 0.964 3 h: 0.966 6 h: 0.968 12 h: 0.964 24 h: 0.958  qSOFA: 0.985–0.998 SOFA: 0.874–0.929 SIRS: 0.936–0.946 MEWS: 0.991–0.995 NEWS: 0.959–0.988 | 1, 3, 6, 12, and 24 hours after the index time | ML model was more timely and discriminant than benchmark screening tools |
| Perng et al. | 72 h / 28 days: CNN + SoftMax: 87.01% RF: 62.56% KNN: 77.31% SVM: 74.33%  SIRS: 59.43% qSOFA: 67.27% |  |  | 72 hours and 28 days post-admission | ML methods were higher than those for existing medical methods like SIRS and qSOFA |
| Kim et al. |  | MARS: 0.732 [0.711–0.753] SVM: 0.736 [0.715–0.756] GBM: 0.722 [0.701–0.743] RF: 0.747 [0.727–0.767] Lasso: 0.716 [0.695–0.737] Ridge: 0.715 [0.693–0.735] Ensemble-Ave: 0.748 [0.727–0.768] Ensemble-MARS: 0.747 [0.727–0.767] MLP: 0.720 [0.698–0.740]  qSOFA: 0.423 [0.401–0.446] MEWS: 0.352 [0.330–0.374] | For ML: 0.900 [0.895–0.904]  qSOFA: 0.950 [0.947–0.953] MEWS: 0.933 [0.929–0.937] | Within 24 hours of ED arrival | ML models significantly outperformed qSOFA and MEWS in screening septic shock |
| Mohamed et al. | NN: 92.2% SVM: 91.0% DT: 91.0% KNN: 90.9% LR: 89.7% | NN: 92.1% SVM: 91.4% DT: 92.1% KNN: 91.1% LR: 88.3% | NN: 92.3% SVM: 90.6% DT: 90.1% KNN: 90.8% LR: 91.1% | within the first 6 hours of ED care | The Neural Network model showed the great performance in identifying sepsis |
| Zhao et al. |  |  |  | 28-day mortality prediction | ML models outperformed traditional scores like SOFA, SAPS II and OASIS for mortality prediction |
| Kwon et al. |  |  |  | Within 3 days of ED admission | ML Model outperformed traditional qSOFA, SIRS and MEWS scores in predicting 3-day mortality |
| Bedoya et al. |  | 1 h, 4 h, 7 h, 10 h MGP–RNN: 0.383–0.644–0.765–0.855 Cox: 0.336–0.580–0.703–0.816 RF: 0.366–0.607–0.698–0.760 PLR: 0.312–0.514–0.656–0.762  SIRS: 0.137–0.375–0.534–0.692 NEWS: 0.069–0.206–0.320–0.445 qSOFA: 0.044–0.163–0.248–0.312 |  | Median of 5 hours before sepsis onset | Models outperformed clinical scores (qSOFA, NEWS, SIRS) |
| Scott et al. |  | Temporal Test: 84% [71%-92%] Geographic Test: 80% [28%-99%] | Temporal Test: 65% [61%-69%] Geographic Test: 40% [27%-54%] | 2 hours since arrival | Model improved early identification of pediatric septic shock |
| van Doorn et al. | XGB: 0.80 [0.72–0.88] LR: 0.83 [0.82–0.83] MLP: 0.87 [0.86–0.88] RF: 0.84 [0.83–0.85]  abbMEDS: 0.70 [0.61–0.79] mREMS: 0.64 [0.55–0.73] SOFA: 0.74 [0.65–0.83] Physician: 0.74 [0.65–0.82] | XGB: 0.92 [0.87–0.95]  abbMEDS: 0.54 [0.44–0.64] mREMS: 0.62 [0.52–0.72] SOFA: 0.77 [0.69–0.85] Phyiscian: 0.72 [0.62–0.81] | XGB: 0.78 [0.70–0.86]  abbMEDS: 0.72 [0.64–0.81] mREMS: 0.64 [0.55–0.74] SOFA: 0.74 [0.65–0.82] Phyiscian: 0.74 [0.64–0.82] | within 2 hours of ED admission | Model outperformed physicians and clinical risk scores like MEDS, REMS, and SOFA |
| Zhang et al. |  |  |  | 4 hours before detection | The proposed model outperformed MEWS, NEWS, SIRS and qSOFA |
| Taneja et al. |  | RF: 0.80  SOFA: 0.81 SIRS: 0.71 | RF: 0.70  SOFA: 0.42 SIRS: 0.80 | 12 hours from ED presentation | Algorithm stratified patients into low, medium and high-risk groups for outcomes like length of stay, 30-day mortality as well as re-admission, outperforming SOFA and SIRS scores |
| Ehwerhemuepha et al. |  | Mortality: 66.67% Severe sepsis: 84.5% Non-severe sepsis: 44.14% | Mortality: 99.5% Severe sepsis: 99.0% Non-severe sepsis: 99.9% | Within a few seconds (5~7 sec) of ED triage | Implementation of the model can predict sepsis-related critical decompensation much earlier than existing tools (e.g., SIRS, qSOFA) |
| Wardi et al. |  | 12h: 0.85 | 12h: 0.678 | Predictions at 8, 12, 16, 24, and 36 hours | Model outperformed traditional methods and demonstrated external generalizability through transfer learning |
| Yun et al. |  | XGB: 0.77 [0.70–0.84] LR: 0.77 [0.70–0.84] ANN: 0.77 [0.70–0.84]  NEWS:  0.65 [0.57–0.73] (Higher threshold) 0.77 [0.70–0.84] (Lower threshold) | XGB: 0.74 [0.73–0.75] LR: 0.74 [0.73–0.75] ANN: 0.74 [0.73–0.75]  NEWS:  0.80 [0.79–0.81] (Higher threshold) 0.70 [0.69–0.71] (Lower threshold) | Within 24 hours of ED arrival | ML models outperformed the NEWS model in predicting septic shock |
| Karlsson et al. |  | 7-day: 0.84 [0.78–0.89] 30-day: 0.87 [0.81–0.93] | 7-day: 0.67 [0.64–0.70] 30-day: 0.64 [0.61–0.67] | Prediction of mortality within 7 and 30 days | RF model performed well using 6 key variables |
| Shashikumar et al. |  | 0.836 | 0.900 | 2.1 [0.8 4.5] hours before antibiotics order | Allowing for identification and prioritization of patients at high risk for sepsis |
| Lin et al. | XGB: 0.78  SIRS: 0.69 qSOFA: 0.79 | XGB: 0.80  SIRS: 0.64 qSOFA: 0.35 | XGB: 0.78  SIRS: 0.66 qSOFA: 0.96 |  | XGBoost outperformed clinical scores (SIRS, qSOFA) |
| Chao et al. |  |  |  | Prediction of 28-day mortality | ML models outperformed traditional models (SOFA, NEWS, SIRS) in predicting mortality |
| Kijpaisalratana et al. |  | RF: 86.94% [82.06%–90.89%] LR: 86.94% [82.06%–90.89%] GB: 84.49% [79.34%–88.78%] NN: 86.94% [82.06%–90.89%]  qSOFA: 28.98% [23.38%–35.10%] MEWS: 49.80% [43.37%–56.23%] SIRS: 84.08% [78.89%–88.43%] | RF: 86.92% [86.51%–87.32%] LR: 87.25% [86.85%–87.65%] GB: 84.80% [84.37%–85.23%] NN: 86.31% [85.89%–86.72%]  qSOFA: 98.10% [97.93%–98.26%] MEWS: 87.71% [87.31%–88.10%] SIRS: 78.67% [78.18%–79.16%] | At triage | ML models significantly outperformed traditional screening tools (qSOFA, MEWS, SIRS) in sepsis detection |
| Choi et al. |  | Fragmented model: 0.710 [0.611–0.792] Accumulated model: 0.699 [0.599–0.783] | Fragmented model: 0.936 [0.907–0.956] Accumulated model: 0.880 [0.844–0.908] | 6 hours since arrival | Wireless wearable devices improved early recognition of clinical deterioration |
| Cheng et al. | CNN: 6 h: 0.905 12 h: 0.872 24 h: 0.860 48 h: 0.828 LSTM: 6 h: 0.817 12 h: 0.759 24 h: 0.789 48 h: 0.759 RF: 6 h: 0.835 12 h: 0.829 24 h: 0.810 48 h: 0.776 | CNN: 6 h: 0.896 12 h: 0.884 24 h: 0.895 48 h: 0.854 LSTM: 6 h: 0.795 12 h: 0.748 24 h: 0.779 48 h: 0.778 RF: 6 h: 0.797 12 h: 0.794 24 h: 0.776 48 h: 0.737 |  | Predict mortality in septic patients within 6–48 h of admission | CNN outperformed classic risk score tools like MEDS, MEWS, REMS and qSOFA, with highest performance at shorter lead times |
| Aguirre et al. | MLP: 0.87 [0.81–0.91] LR: 0.80 [0.74–0.86] NB: 0.81 [0.75–0.86] KNN: 0.85 [0.79–0.89] SVM: 0.87 [0.81–0.91] RF: 0.87 [0.82–0.91] XGB: 0.84 [0.79–0.89] | MLP: 0.85 [0.76–0.92] LR: 0.62 [0.51–0.73] NB: 0.74 [0.64–0.83] KNN: 0.76 [0.65–0.84] SVM: 0.91 [0.83–0.96] RF: 0.84 [0.74–0.91] XGB: 0.79 [0.69–0.87] | MLP: 0.88 [0.81–0.93] LR: 0.92 [0.86–0.96] NB: 0.86 [0.79–0.91] KNN: 0.91 [0.84–0.95] SVM: 0.84 [0.76–0.90] RF: 0.89 [0.82–0.94] XGB: 0.88 [0.80–0.93] |  | Model showed the excellent performance in terms of calibration, clinical usefulness |
| Chiu et al. | 0.82 | 0.83 | 0.82 |  | Model outperformed traditional SOFA model |
| Mercurio et al. |  | LR: 0.76 RF: 0.93 CART: 0.85 SVM: 0.70 GNB: 0.37 | LR: 0.88 RF: 0.84 CART: 0.70 SVM: 0.92 GNB: 0.94 |  | Identified sepsis predictors for best models' performance |
| Greco et al. | RF: 0.766 LR: 0.844 | RF: 0.769 LR: 0.972 |  |  | RF outperformed clinical scores (qSOFA, SOFA, APACHE II) in predicting mortality |
| Guo et al. |  |  |  | within 72 hours of ED admission | EASE (LR) model outperformed NEWS and showed good calibration |
| Jeon et al. | LightGBM:  7-day: 0.78 14-day: 0.77 30-day: 0.79 XGBoost: 7-day: 0.76 14-day: 0.77 30-day: 0.74 SVM: 7-day: 0.70 14-day: 0.73 30-day: 0.76 MLP: 7-day: 0.79 14-day: 0.78 30-day: 0.78 LR: 7-day: 0.66 14-day: 0.65 30-day: 0.72 |  |  | Prediction of 7-day, 14-day and 30-day mortality | ML outperformed traditional scoring systems (SOFA, qSOFA, NEWS, NEWS2, MEWS) in predicting sepsis mortality |
| Prasad et al. | Lower threshold (sensitivity > 80%): 68% Higher threshold (specificity > 80%): 72% | Lower threshold (sensitivity > 80%): 80% Higher threshold (specificity > 80%): 36% | Lower threshold (sensitivity > 80%): 68% Higher threshold (specificity > 80%): 72% | At triage | Model improved sepsis detection performance compared to qSOFA |
| Wong et al. | ANN: 87.1% | qSOFA: 56.39% | qSOFA: 74.58% | 30 days since arrival | Model better discriminatory performance in predicting 30-day mortality than qSOFA |
| Brann et al. |  | Time-of-Triage Model: 0.85 Comprehensive Model: 1h: 0.72 5h: 0.87 12h: 0.92 | Time-of-Triage Model: 0.86 Comprehensive Model: 1h: 0.94 5h: 0.91 12h: 0.89 | up to 12 hours before | NLP-based ML model improved timeliness of sepsis identification |
| Park et al. | CAT: 0.769 [0.742–0.798] LR: 0.731 [0.693–0.769] SVM: 0.729 [0.691–0.767] RF: 0.701 [0.661–0.740] XGB: 0.773 [0.744–0.802] LGB: 0.763 [0.726–0.797] | CAT: 0.347 [0.269–0.428] LR: 0.667 [0.586–0.738] SVM: 0.694 [0.621–0.766] RF: 0.584 [0.503–0.662] XGB: 0.381 [0.303–0.462] LGB: 0.516 [0.435–0.600] | CAT: 0.937 [0.910–0.962] LR: 0.757 [0.713–0.801] SVM: 0.743 [0.697–0.787] RF: 0.747 [0.702–0.792] XGB: 0.929 [0.902–0.954] LGB: 0.861 [0.825–0.896] | 12 hours of ED admission | ML models significantly outperformed SOFA score in predicting mortality |
| Aygun et al. | XGB: 0.898 [0.868–0.929] AdaBoost: 0.869 [0.835–0.903] LightGBM: 0.888 [0.856–0.92] | XGB: 0.905 [0.854–0.943] AdaBoost: 0.882 [0.827–0.925] LightGBM: 0.895 [0.842–0.935] | XGB: 0.891 [0.837–0.932] AdaBoost: 0.856 [0.797–0.903] LightGBM: 0.880 [0.825–0.924] | At admission to ED | Models identified key biomarkers for early sepsis diagnosis with high accuracy |
| Xia et al. | RF: 0.836 | RF: 0.810 | RF: 0.906 | Within 24 hours of admission | RF model outperformed traditional scoring systems (qSOFA, APACHE II, BISAP) in predicting sepsis in AP patients |
| Hou et al. |  | 86.90% | 92.50% | Updates every 5 minutes during ED stay | Model outperformed conventional tools like SIRS, qSOFA, SOFA and NEWS in sepsis detection |
| Xie et al. | 84.1% [83.8%–84.4%] | 84.4% [84.1%–84.7%] | 83.7% [83.4%–84.0%] | within 24 hours of ED admission | ML could be applied to effectively predict sepsis |
| Song et al. | XGBoost: 0.80 [0.80–0.80] LR: 0.78 [0.78–0.78] RF: 0.79 [0.79–0.79] AutoScore: 0.81 [0.81–0.81]  NEWS: 0.78 [0.78–0.78] NEWS2: 0.81 [0.81–0.81] MEWS: 0.75 [0.75–0.75] CART: 0.72 [0.72–0.72] REMS: 0.60 [0.60–0.60] ESI: 0.63 [0.63–0.63] | XGBoost: 0.80 [0.80–0.80] LR: 0.85 [0.83–0.86] RF: 0.84 [0.83–0.85] AutoScore: 0.80 [0.79–0.82]  NEWS: 0.53 [0.51–0.55] NEWS2: 0.48 [0.46–0.50] MEWS: 0.50 [0.48–0.52] CART: 0.64 [0.63–0.66] REMS: 0.73 [0.71–0.75] ESI: 0.87 [0.86–0.88] | XGBoost: 0.84 [0.82–0.85] LR: 0.78 [0.77–0.80] RF: 0.79 [0.79–0.79] AutoScore: 0.81 [0.81–0.81]  NEWS: 0.79 [0.79–0.79] NEWS2: 0.82 [0.82–0.82] MEWS: 0.75 [0.75–0.75] CART: 0.72 [0.72–0.72] REMS: 0.60 [0.60–0.60] ESI: 0.62 [0.61–0.62] |  | ML models outperformed other scoring systems for the early prediction of sepsis among patients in ED |

##### Supplementary 4: Quality Assessment

###### 4.1 Quality Assessment Standards

**Table S7. Quality assessment standard.**

| **Section** | **Topic** | **Item** | **Checklist item** |  |
| --- | --- | --- | --- | --- |
|  |  |  |  |  |
|  | Title | 1 | Identify the study as developing or evaluating the performance of a multivariable prediction model, the target population, and the outcome to be predicted |  |
|  | Abstract | 2 | Structured abstract includes background, objective, method, result, discussion, registration and so on |  |
| Introduction | Healthcare Background | 3 | Explain the healthcare context (including whether diagnostic or prognostic) and rationale for developing or evaluating the prediction model, including references to existing models |  |
| Introduction | Targeted Population | 4 | Describe the target population and the intended purpose of the prediction model in the context of the care pathway, including its intended users (eg, healthcare professionals, patients, public) |  |
| Introduction | Health Inequalities | 5 | Describe any known health inequalities between sociodemographic groups |  |
| Introduction | Study Objectives | 6 | Specify the study objectives, including whether the study describes the development or validation of a prediction model (or both) |  |
| Methods | Data Sources | 7 | Describe the sources of data separately for the development and evaluation datasets (eg, randomised trial, cohort, routine care or registry data), the rationale for using these data, and representativeness of the data |  |
| Methods | Collection Period | 8 | Specify the dates of the collected participant data, including start and end of participant accrual; and, if applicable, end of follow-up |  |
| Methods | Study Setting | 9 | Specify key elements of the study setting (eg, primary care, secondary care, general population) including the number and location of centres |  |
| Methods | Eligibility Criteria | 10 | Describe the eligibility criteria for study participants |  |
| Methods | Treatments Received | 11 | Give details of any treatments received, and how they were handled during model development or evaluation, if relevant |  |
| Methods | Data Pre-Processing | 12 | Describe any data pre-processing and quality checking, including whether this was similar across relevant sociodemographic groups |  |
| Methods | Outcome Definition | 13 | Clearly define the outcome that is being predicted and the time horizon, including how and when assessed, the rationale for choosing this outcome, and whether the method of outcome assessment is consistent across sociodemographic groups |  |
| Methods | Subjective Outcome | 14 | If outcome assessment requires subjective interpretation, describe the qualifications and demographic characteristics of the outcome assessors |  |
| Methods | Blind Assessment of Outcomes | 15 | Report any actions to blind assessment of the outcome to be predicted |  |
| Methods | Initial Predictors | 16 | Describe the choice of initial predictors (eg, literature, previous models, all available predictors) and any pre-selection of predictors before model building |  |
| Methods | Define Predictors | 17 | Clearly define all predictors, including how and when they were measured (and any actions to blind assessment of predictors for the outcome and other predictors) |  |
| Methods | Subjective Predictor | 18 | If predictor measurement requires subjective interpretation, describe the qualifications and demographic characteristics of the predictor assessors |  |
| Methods | Sample Size Justification | 19 | Explain how the study size was arrived at (separately for development and evaluation), and justify that the study size was sufficient to answer the research question. Include details of any sample size calculation |  |
| Methods | Missing Data Handling | 20 | Describe how missing data were handled. Provide reasons for omitting any data |  |
| Methods | Data Partitioning | 21 | Describe how the data were used (eg, for development and evaluation of model performance) in the analysis, including whether the data were partitioned, considering any sample size requirements |  |
| Methods | Predictor Handling | 22 | Depending on the type of model, describe how predictors were handled in the analyses (functional form, rescaling, transformation, or any standardisation) |  |
| Methods | Model Building | 23 | Specify the type of model, rationale, all model building steps, including any hyperparameter tuning, and method for internal validation |  |
| Methods | Estimate Heterogeneity | 24 | Describe if and how any heterogeneity in estimates of model parameter values and model performance was handled and quantified across clusters (eg, hospitals, countries). See TRIPOD-Cluster for additional considerations |  |
| Methods | Performance Measures | 25 | Specify all measures and plots used (and their rationale) to evaluate model performance (eg, discrimination, calibration, clinical utility) and, if relevant, to compare multiple models |  |
| Methods | Model Updating | 26 | Describe any model updating (eg, recalibration) arising from the model evaluation, either overall or for particular sociodemographic groups or settings |  |
| Methods | Calculated Predictions | 27 | For model evaluation, describe how the model predictions were calculated (eg, formula, code, object, application programming interface) |  |
| Methods | Class Imbalance | 28 | If class imbalance methods were used, state why and how this was done, and any subsequent methods to recalibrate the model or the model predictions |  |
| Methods | Fairness Assessment | 29 | Describe any approaches that were used to address model fairness and their rationale |  |
| Methods | Model Output | 30 | Specify the output of the prediction model (eg, probabilities, classification). Provide details and rationale for any classification and how the thresholds were identified |  |
| Methods | Training vs. Evaluation | 31 | Identify any differences between the development and evaluation data in healthcare setting, eligibility criteria, outcome, and predictors |  |
| Methods | Ethical Approval | 32 | Name the institutional research board or ethics committee that approved the study and describe the participant informed consent or the ethics committee waiver of informed consent |  |
| Open Science | Funding | 33 | Give the source of funding and the role of the funders for the present study |  |
| Open Science | Conflicts of Interest | 34 | Declare any conflicts of interest and financial disclosures for all authors |  |
| Open Science | Protocol | 35 | Indicate where the study protocol can be accessed or state that a protocol was not prepared |  |
| Open Science | Registration | 36 | Provide registration information for the study, including register name and registration number, or state that the study was not registered |  |
| Open Science | Data Availability | 37 | Provide details of the availability of the study data |  |
| Open Science | Code Availability | 38 | Provide details of the availability of the analytical code |  |
|  | Patient/Public Involvement | 39 | Provide details of any patient and public involvement during the design, conduct, reporting, interpretation, or dissemination of the study or state no involvement |  |
| Result | Participant Flow | 40 | Describe the flow of participants through the study, including the number of participants with and without the outcome and, if applicable, a summary of the follow-up time. A diagram may be helpful |  |
| Result | Report Characteristics | 41 | Report the characteristics overall and, where applicable, for each data source or setting, including the key dates, key predictors (including demographics), treatments received, sample size, number of outcome events, follow-up time, and amount of missing data. A table may be helpful. Report any differences across key demographic groups |  |
| Result | Predictor Distribution | 42 | For model evaluation, show a comparison with the development data of the distribution of important predictors (demographics, predictors, and outcome) |  |
| Result | Model Development | 43 | Specify the number of participants and outcome events in each analysis (eg, for model development, hyperparameter tuning, model evaluation) |  |
| Result | Model Specification | 44 | Provide details of the full prediction model (eg, formula, code, object, application programming interface) to allow predictions in new individuals and to enable third party evaluation and implementation, including any restrictions to access or reuse (eg, freely available, proprietary) |  |
| Result | Performance Estimates | 45 | Report model performance estimates with confidence intervals, including for any key subgroups (eg, sociodemographic). Consider plots to aid presentation |  |
| Result | Performance Heterogeneity | 46 | If examined, report results of any heterogeneity in model performance across clusters. See TRIPOD-Cluster for additional details |  |
| Result | Model Monitoring | 47 | Report the results from any model updating, including the updated model and subsequent performance |  |
| Discussion | Interpretation | 48 | Give an overall interpretation of the main results, including issues of fairness in the context of the objectives and previous studies |  |
| Discussion | Limitation | 49 | Discuss any limitations of the study (such as a non-representative sample, sample size, overfitting, missing data) and their effects on any biases, statistical uncertainty, and generalisability |  |
| Discussion | Input Data Quality | 50 | Describe how poor quality or unavailable input data (eg, predictor values) should be assessed and handled when implementing the prediction model |  |
| Discussion | User Interaction | 51 | Specify whether users will be required to interact in the handling of the input data or use of the model, and what level of expertise is required of users |  |
| Discussion | Future Research | 52 | Discuss any next steps for future research, with a specific view to applicability and generalisability of the model |  |

###### 4.2 Quality Assessment Results

**Table S8. Quality assessment results (Part A).**

| Topic | Delahanty et al. | Perng et al. | Kim et al. | Mohamed et al. | Zhao et al. | Kwon et al. | Bedoya et al. | Scott et al. | van Doorn et al. | Zhang et al. | Taneja et al. | Ehwerhemuepha et al. |
| --- | --- | --- | --- | --- | --- | --- | --- | --- | --- | --- | --- | --- |
| Title | √ | √ | × | √ | √ | √ | × | × | √ | × | × | √ |
| Abstract | √ | √ | √ | √ | √ | √ | √ | √ | √ | √ | √ | √ |
| Healthcare Background | √ | √ | √ | √ | √ | √ | √ | √ | √ | √ | √ | √ |
| Targeted Population | √ | √ | √ | √ | √ | √ | √ | √ | √ | √ | √ | √ |
| Health Inequalities | × | × | × | × | × | × | × | × | × | × | × | × |
| Study Objectives | √ | √ | √ | √ | √ | √ | √ | √ | √ | √ | √ | √ |
| Data Sources | √ | √ | √ | √ | √ | √ | √ | √ | √ | √ | √ | √ |
| Collection Period | √ | √ | √ | × | √ | √ | √ | √ | √ | √ | √ | √ |
| Study Setting | √ | √ | √ | √ | √ | √ | √ | √ | √ | √ | √ | √ |
| Eligibility Criteria | √ | √ | √ | √ | √ | √ | √ | √ | √ | √ | √ | √ |
| Treatments Received |  | √ |  |  | √ |  | √ |  |  |  |  | √ |
| Data Pre-Processing | √ | √ | √ | √ | √ | √ | √ | √ | √ | √ | √ | √ |
| Outcome Definition | √ | √ | √ | √ | √ | √ | √ | √ | √ | √ | √ | √ |
| Subjective Outcome |  |  |  | × |  |  |  |  |  |  |  |  |
| Blind Assessment of Outcomes |  |  |  | × |  |  |  |  |  |  |  |  |
| Initial Predictors | √ | √ | √ | √ | √ | √ | √ | √ | √ | √ | √ | √ |
| Define Predictors | √ | √ | √ | √ | √ | √ | √ | √ | √ | √ | √ | √ |
| Subjective Predictor |  | × | × | × |  | × |  | × | × |  |  | × |
| Sample Size Justification | × | × | × | × | × | × | × | × | × | × | × | × |
| Missing Data Handling | √ | √ | √ | √ | × | × | √ | √ | √ | √ | √ | √ |
| Data Partitioning | √ | √ | √ | √ | √ | √ | √ | √ | √ | √ | √ | √ |
| Predictor Handling | √ | √ | √ | √ | √ | √ | √ | √ | √ | √ | √ | √ |
| Model Building | √ | √ | √ | √ | √ | √ | √ | √ | √ | √ | √ | √ |
| Estimate Heterogeneity | × |  |  |  |  | × |  | × |  |  | × |  |
| Performance Measures | √ | √ | √ | √ | √ | √ | √ | √ | √ | √ | √ | √ |
| Model Updating | × | × | × | × | × | × | × | × | × | × | × | × |
| Calculated Predictions | × | × | × | × | × | × | × | √ | × | √ | × | × |
| Class Imbalance |  |  |  |  |  | √ |  |  |  |  |  | √ |
| Fairness Assessment | × | × | × | × | × | × | × | × | × | √ | × | × |
| Model Output | √ | √ | √ | √ | √ | √ | √ | √ | √ | √ | √ | √ |
| Training vs. Evaluation | √ | × | × | × | √ | √ | √ | √ | √ | × | √ | × |
| Ethical Approval | √ | √ | √ | × | √ | √ | √ | √ | √ | √ | √ | √ |
| Funding | √ | √ | √ | × | √ | √ | √ | √ | √ | √ | √ | × |
| Conflicts of Interest | √ | √ | √ | × | √ | √ | √ | √ | √ | √ | √ | √ |
| Protocol | × | × | × | × | × | × | × | × | × | × | × | × |
| Registration | × | × | × | × | × | × | × | × | × | × | × | × |
| Data Availability | × | × | × | × | √ | × | × | × | √ | × | × | × |
| Code Availability | × | × | × | × | × | × | × | × | × | √ | × | × |
| Patient/Public Involvement | × | × | × | × | × | × | × | × | × | × | × | × |
| Participant Flow | √ | √ | √ | × | √ | √ | √ | √ | √ | √ | √ | × |
| Report Characteristics | √ | √ | √ | √ | √ | √ | √ | √ | √ | √ | √ | √ |
| Predictor Distribution | √ | √ | √ | × | √ | × | × | × | × | × | √ | √ |
| Model Development | √ | √ | √ | √ | √ | √ | √ | √ | √ | √ | √ | √ |
| Model Specification | × | × | × | × | × | × | × | √ | × | × | × | × |
| Performance Estimates | × | √ | √ | × | √ | √ | √ | √ | √ | × | × | √ |
| Performance Heterogeneity |  |  |  |  |  | × |  | × |  |  | × |  |
| Model Monitoring | × | × | × | × | × | × | × | × | × | × | × | × |
| Interpretation | √ | √ | √ | √ | √ | √ | √ | √ | √ | √ | √ | √ |
| Limitation | √ | √ | √ | × | √ | √ | √ | √ | √ | √ | √ | √ |
| Input Data Quality | × | × | × | × | × | × | × | × | × | × | × | × |
| User Interaction | × | × | × | × | × | × | × | × | × | × | × | × |
| Future Research | √ | √ | √ | × | √ | √ | √ | √ | √ | √ | √ | √ |

**Table S8. Quality assessment results (Part B).**

| Topic | Wardi et al. | Yun et al. | Karlsson et al. | Shashikumar et al. | Lin et al. | Chao et al. | Kijpaisalratana et al. | Choi et al. | Cheng et al. | Aguirre et al. | Chiu et al. | Mercurio et al. |
| --- | --- | --- | --- | --- | --- | --- | --- | --- | --- | --- | --- | --- |
| Title | × | × | √ | × | √ | √ | × | √ | √ | × | √ | × |
| Abstract | √ | √ | √ | √ | √ | √ | √ | √ | √ | √ | √ | √ |
| Healthcare Background | √ | √ | √ | √ | √ | √ | √ | √ | √ | √ | √ | √ |
| Targeted Population | √ | √ | √ | √ | √ | √ | √ | √ | √ | √ | √ | √ |
| Health Inequalities | × | × | × | × | × | × | × | × | × | × | × | √ |
| Study Objectives | √ | √ | √ | √ | √ | √ | √ | √ | √ | √ | √ | √ |
| Data Sources | √ | √ | √ | √ | √ | √ | √ | √ | √ | √ | √ | √ |
| Collection Period | √ | √ | √ | √ | √ | √ | √ | √ | √ | √ | √ | √ |
| Study Setting | √ | √ | √ | √ | √ | √ | √ | √ | √ | √ | √ | √ |
| Eligibility Criteria | √ | √ | √ | √ | √ | √ | √ | √ | √ | √ | √ | √ |
| Treatments Received | × | × | × | × | × | × | × | × | × | × | √ | √ |
| Data Pre-Processing | √ | √ | √ | √ | √ | √ | √ | √ | √ | √ | √ | √ |
| Outcome Definition | √ | √ | √ | √ | √ | √ | √ | √ | √ | √ | √ | √ |
| Subjective Outcome |  |  |  |  |  |  |  |  |  |  |  |  |
| Blind Assessment of Outcomes |  |  |  |  |  |  |  |  |  |  |  |  |
| Initial Predictors | √ | √ | √ | √ | √ | √ | √ | √ | √ | √ | √ | √ |
| Define Predictors | √ | √ | √ | √ | √ | √ | √ | √ | √ | √ | √ | √ |
| Subjective Predictor |  | × | × |  | × | × | × | × |  |  | × | × |
| Sample Size Justification | × | × | × | √ | × | √ | × | × | × | × | × | × |
| Missing Data Handling | √ | √ | √ | √ | √ | √ | √ | √ | √ | × | √ | √ |
| Data Partitioning | √ | √ | √ | √ | √ | √ | √ | √ | √ | √ | √ | √ |
| Predictor Handling | √ | √ | √ | √ | √ | √ | √ | √ | √ | √ | √ | √ |
| Model Building | √ | √ | √ | √ | √ | √ | √ | √ | √ | √ | √ | √ |
| Estimate Heterogeneity | × |  |  | × | × | × |  |  | × |  |  |  |
| Performance Measures | √ | √ | √ | √ | √ | √ | √ | √ | √ | √ | √ | √ |
| Model Updating | √ | × | × | × | × | × | √ | × | × | × | × | × |
| Calculated Predictions | × | × | × | × | × | × | × | × | × | × | × | × |
| Class Imbalance |  |  | √ |  |  | √ | √ |  | √ |  | √ | √ |
| Fairness Assessment | × | × | × | × | × | × | × | × | × | × | × | √ |
| Model Output | √ | √ | √ | √ | √ | √ | √ | √ | √ | √ | √ | √ |
| Training vs. Evaluation | √ | √ | × | √ | √ | × | × | × | × | √ | √ | × |
| Ethical Approval | √ | √ | √ | √ | √ | √ | √ | √ | √ | √ | √ | √ |
| Funding | √ | √ | √ | √ | √ | √ | √ | √ | × | × | × | √ |
| Conflicts of Interest | √ | × | √ | √ | √ | √ | × | √ | √ | × | × | √ |
| Protocol | × | × | × | × | × | × | × | × | × | × | × | × |
| Registration | × | × | × | × | × | × | × | × | × | × | × | × |
| Data Availability | × | × | × | √ | × | × | × | × | × | × | × | × |
| Code Availability | × | × | √ | √ | × | × | × | × | × | × | × | × |
| Patient/Public Involvement | × | × | × | × | × | × | × | × | × | × | × | × |
| Participant Flow | √ | √ | √ | × | × | × | √ | √ | × | √ | √ | √ |
| Report Characteristics | √ | √ | √ | √ | √ | √ | √ | √ | × | √ | √ | √ |
| Predictor Distribution | × | √ | √ | × | × | √ | √ | × | × | √ | √ | √ |
| Model Development | √ | √ | √ | √ | √ | √ | √ | √ | √ | √ | √ | √ |
| Model Specification | × | × | × | × | × | × | × | × | × | × | × | × |
| Performance Estimates | × | √ | √ | × | × | √ | √ | √ | × | √ | × | × |
| Performance Heterogeneity | × |  |  | × | × | × |  |  | × |  |  |  |
| Model Monitoring | × | × | × | × | × | × | × | × | × | × | × | × |
| Interpretation | √ | √ | √ | √ | √ | √ | √ | √ | √ | √ | √ | √ |
| Limitation | √ | √ | √ | √ | √ | √ | √ | √ | √ | √ | × | √ |
| Input Data Quality | × | × | × | √ | × | × | × | × | × | × | × | × |
| User Interaction | × | × | × | × | × | × | × | × | × | × | × | × |
| Future Research | √ | √ | √ | √ | √ | √ | √ | √ | √ | √ | × | √ |

**Table S8. Quality assessment results (Part C).**

| Topic | Greco et al. | Guo et al. | Jeon et al. | Prasad et al. | Wong et al. | Brann et al. | Park et al. | Aygun et al. | Xia et al. | Hou et al. | Xie et al. | Song et al. |
| --- | --- | --- | --- | --- | --- | --- | --- | --- | --- | --- | --- | --- |
| Title | √ | √ | √ | × | × | × | √ | √ | √ | √ | × | √ |
| Abstract | √ | √ | √ | √ | √ | √ | √ | √ | √ | √ | √ | √ |
| Healthcare Background | √ | √ | √ | √ | √ | √ | √ | √ | √ | √ | √ | √ |
| Targeted Population | √ | √ | √ | √ | √ | √ | √ | √ | √ | √ | √ | √ |
| Health Inequalities | × | × | × | × | × | × | × | × | × | × | × | × |
| Study Objectives | √ | √ | √ | √ | √ | √ | √ | √ | √ | √ | √ | √ |
| Data Sources | √ | √ | √ | √ | √ | √ | √ | √ | √ | √ | √ | √ |
| Collection Period | × | √ | √ | √ | √ | √ | √ | × | √ | √ | √ | √ |
| Study Setting | √ | √ | √ | √ | √ | √ | √ | × | √ | √ | √ | √ |
| Eligibility Criteria | √ | √ | √ | √ | √ | √ | √ | √ | √ | √ | √ | √ |
| Treatments Received | × | × | × | × | × | × | √ | × | × | × | × | × |
| Data Pre-Processing | √ | √ | √ | √ | √ | √ | √ | √ | √ | √ | √ | √ |
| Outcome Definition | √ | √ | √ | √ | √ | √ | √ | √ | √ | √ | √ | √ |
| Subjective Outcome |  |  |  |  |  |  |  |  |  |  |  |  |
| Blind Assessment of Outcomes |  |  |  |  |  |  |  |  |  |  |  |  |
| Initial Predictors | √ | √ | √ | √ | √ | √ | √ | √ | √ | √ | √ | √ |
| Define Predictors | √ | √ | √ | √ | √ | √ | √ | √ | √ | √ | √ | √ |
| Subjective Predictor | × | × | × | √ |  | × | × |  | × | × | × | × |
| Sample Size Justification | × | × | × | √ | × | × | × | × | × | × | × | × |
| Missing Data Handling | √ | √ | √ | √ | √ | √ | √ | √ | √ | √ | √ | √ |
| Data Partitioning | √ | √ | √ | √ | √ | √ | √ | √ | √ | √ | √ | √ |
| Predictor Handling | × | √ | √ | √ | √ | √ | √ | × | × | √ | √ | √ |
| Model Building | √ | √ | √ | √ | √ | √ | √ | √ | √ | √ | √ | √ |
| Estimate Heterogeneity |  | × |  | × |  | × | × |  | × |  |  |  |
| Performance Measures | √ | √ | √ | √ | √ | √ | √ | √ | √ | √ | √ | √ |
| Model Updating | × | × | √ | × | × | × | × | × | × | × | × | √ |
| Calculated Predictions | × | × | × | × | × | × | × | × | × | × | × | × |
| Class Imbalance | √ |  |  |  |  | √ |  | √ | √ |  | √ |  |
| Fairness Assessment | × | × | × | √ | × | × | × | × | × | × | × | × |
| Model Output | √ | √ | √ | √ | √ | √ | √ | √ | √ | √ | √ | √ |
| Training vs. Evaluation | × | √ | × | √ | × | × | √ | × | × | × | × | × |
| Ethical Approval | √ | √ | √ | √ | √ | √ | √ | √ | √ | √ | √ | √ |
| Funding | √ | √ | √ | √ | √ | × | √ | √ | √ | √ | √ | √ |
| Conflicts of Interest | √ | √ | √ | √ | √ | √ | √ | √ | √ | √ | √ | × |
| Protocol | × | × | × | × | × | × | × | × | × | × | × | × |
| Registration | × | × | × | × | × | × | × | × | × | × | × | × |
| Data Availability | × | × | × | √ | × | × | × | × | × | × | × | × |
| Code Availability | × | × | × | √ | × | × | × | × | × | × | × | × |
| Patient/Public Involvement | × | × | × | × | × | × | × | × | × | × | × | × |
| Participant Flow | √ | √ | √ | √ | √ | × | √ | × | √ | √ | √ | √ |
| Report Characteristics | √ | √ | √ | √ | √ | √ | √ | √ | √ | √ | √ | √ |
| Predictor Distribution | √ | √ | √ | √ | √ | × | √ | √ | × | √ | √ | √ |
| Model Development | × | √ | × | √ | × | √ | √ | × | √ | × | × | × |
| Model Specification | × | × | × | × | × | × | × | × | × | × | × | × |
| Performance Estimates | × | √ | √ | √ | × | × | √ | √ | √ | √ | √ | √ |
| Performance Heterogeneity |  | × |  | × |  | √ | × |  | × |  |  |  |
| Model Monitoring | × | × | × | × | × | × | × | × | × | √ | × | × |
| Interpretation | √ | √ | √ | √ | √ | √ | √ | √ | √ | √ | √ | √ |
| Limitation | √ | √ | √ | √ | √ | √ | √ | × | √ | √ | √ | √ |
| Input Data Quality | × | × | × | × | × | × | × | × | × | √ | × | × |
| User Interaction | × | × | × | × | × | × | × | × | × | √ | × | × |
| Future Research | √ | √ | √ | √ | √ | √ | √ | × | √ | √ | √ | √ |

##### Supplementary 5: Meta-analysis results

###### 5.1 Reported AUROCs of traditional rule-based methods

**Table S9. Performance of traditional rule-based methods.**

| **Author** | **SIRS** | **SOFA** | **qSOFA** | **MEWS** | **NEWS** | **REMS** | **MEDS** |
| --- | --- | --- | --- | --- | --- | --- | --- |
| Delahanty et al. | 0.77 | 0.84 | 0.71 | 0.71 | 0.77 |  |  |
| Bedoya et al. | 0.76 |  | 0.48 |  | 0.62 |  |  |
| Zhang et al. | 0.62 |  | 0.59 | 0.63 | 0.62 |  |  |
| Taneja et al. | 0.83 | 0.71 |  |  |  |  |  |
| Lin et al. | 0.68 |  | 0.56 |  |  |  |  |
| Kijpaisalratana et al. | 0.81 |  | 0.64 | 0.69 |  |  |  |
| Guo et al. |  |  |  |  | 0.78 |  |  |
| Prasad et al. |  |  | 0.63 |  |  |  |  |
| Xia et al. |  |  | 0.67 |  |  |  |  |
| Hou et al. | 0.83 | 0.83 | 0.74 |  | 0.84 |  |  |
| Song et al. |  |  |  | 0.64 | 0.67 | 0.71 |  |
| Perng et al. | 0.63 |  | 0.71 |  |  |  |  |
| Kim et al. |  |  | 0.81 | 0.79 |  |  |  |
| Zhao et al. |  | 0.68 |  |  |  |  |  |
| Kwon et al. | 0.68 |  | 0.78 | 0.77 |  |  |  |
| van Doorn et al. |  | 0.75 |  |  |  | 0.63 | 0.63 |
| Yun et al. |  |  |  |  | 0.80 |  |  |
| Chao et al. |  | 0.75 |  |  |  |  |  |
| Cheng et al. |  |  | 0.67 | 0.66 |  | 0.71 | 0.80 |
| Chiu et al. |  | 0.65 |  |  |  |  |  |
| Greco et al. |  | 0.71 | 0.71 |  |  |  |  |
| Jeon et al. |  | 0.65 | 0.57 | 0.59 | 0.63 |  |  |
| Wong et al. |  |  | 0.69 |  |  |  |  |

###### 5.2 Funnel plot for publication bias test


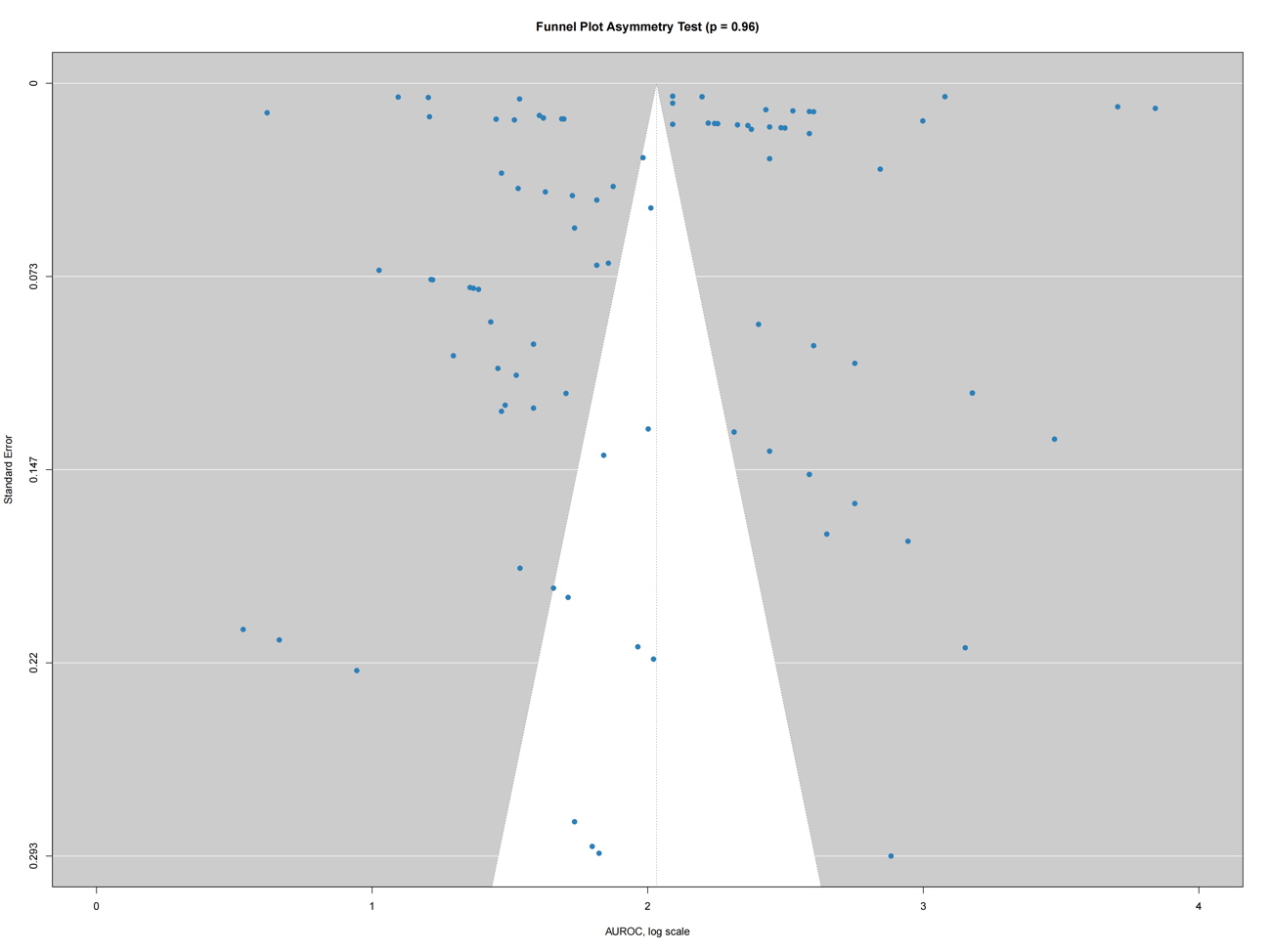


**Fig S1. Funnel plot for publication bias test.**

##### Supplementary 6: Certainty of evidence assessment using GRADEpro GDT

**Table S10. GRADEpro GDT assessment for AI-based sepsis prediction performance by prediction outcome.**

| **Certainty assessment** | | | | | | **Effect** | **Certainty** | **Importance** |
| --- | --- | --- | --- | --- | --- | --- | --- | --- |
| **No. of models** | **Risk of bias** | **Inconsistency** | **Indirectness** | **Imprecision** | **Publication bias** | **Pooled AUROC (95% CI)** |  |  |
| **Early-onset Prediction** | | | | | | | | |
| 44 | Seriousᵃ | Seriousᵇ | Not Serious | Not Serious | Not Detected (t=-0.438, p=0.664) | 0.896 (0.877–0.916) | ⨁⨁〇〇 Low | Critical |
| **Septic Shock Prediction** | | | | | | | | |
| 17 | Seriousᵃ | Seriousᵇ | Not Serious | Not Serious | Not Detected (t=0.503, p=0.622) | 0.882 (0.850–0.914) | ⨁⨁〇〇 Low | Critical |
| **Prognosis Prediction** | | | | | | | | |
| 1 | Seriousᵃ | Not Assessableᵈ | Not Serious | Very Seriousᶜ | Not Assessableᵈ | 0.850 (0.723–0.977) | ⨁〇〇〇 Very Low | Important |
| **Mortality Prediction** | | | | | | | | |
| 36 | Seriousᵃ | Seriousᵇ | Not Serious | Not Serious | Not Detected (t=0.785, p=0.438) | 0.828 (0.806–0.851) | ⨁⨁〇〇 Low | Important |

ᵃ Risk of Bias (Serious): Reporting of items related to potential optimism (e.g., model specification and training-evaluation separation) and external validation is limited.

ᵇ Inconsistency (Serious): High heterogeneity between models is expected due to clinical/methodological diversity.

ᶜ Imprecision (Very Serious): Prognosis evidence is based on a single model/study (k=1) and the confidence interval is wide.

ᵈ Not Assessable: For prognosis (k=1), inconsistency and publication bias cannot be assessed.
